# Automated segmentation of postsurgical resection cavities on magnetic resonance imaging in focal epilepsy: A Multicentre Epilepsy Lesion Detection study

**DOI:** 10.1002/epi.70450

**Published:** 2026-08-19

**Authors:** Jieun Seo, Mathilde Ripart, Helene Kaas, Cornelius Kronlage, Ben Sinclair, Lucy Vivash, Merran R. Courtney, Terence J. O'Brien, Siby Gopinath, Harilal Parasuram, Sedat Kandemirli, Natally Alarab, Lillian Lai, Marcus Likeman, Kai Zhang, Jiajie Mo, Georgian Ciobotaru, James Galea, Philip Sequeiros‐Peggs, Khalid Hamandi, Hua Xie, Venkata Sita Priyanka Illapani, William D. Gaillard, Nathan T. Cohen, Alexander G. Weil, Florence Henrichon‐Goulet, Kenza S. Lahlou, Aristides Hadjinicolaou, Agustín Ibáñez, Gonzalo M. Rojas‐Costa, Horst Urbach, Lara Bücheler, Marcel Heers, Adrián Valls Carbó, Rafael Toledano, Giulia Nobile, Costanza Parodi, Domenico Tortora, Alessandro Consales, Antonella Riva, Mariasavina Severino, Martin Tisdall, Felice D'Arco, Kshitij Mankad, Aswin Chari, Maria H. Eriksson, Rory J. Piper, J. Helen Cross, Torsten Baldeweg, Sofia González‐Ortiz, Jose Pariente, Nuria Bargalló, Yawu Liu, Reetta Kälviäinen, Carmen Barba, Matteo Lenge, Renzo Guerrini, Masaki Iwasaki, Daichi Sone, Hiroyuki Maki, Tomoki Imokawa, Noriko Sato, Julien Jung, Francisco Sepulveda, Daniel Mansilla, Andres Goycoolea, Ingeborg Lopez, Antonio Napolitano, Alessandro De Benedictis, Luca De Palma, Maria Camilla Rossi‐Espagnet, Nikolaos Kondylidis, Kostakis Gkiatis, Kyriakos Garganis, Joshua Pepper, Stefano Seri, John S. Duncan, Clarissa L. Yasuda, Lucas Scárdua‐Silva, Marina K. M. Alvim, Fernando Cendes, Antonio G. Gennari, Ruth O'Gorman Tuura, Georgia Ramantani, Mariam Josyula, Joel Stein, Nishant Sinha, Kate Davis, R. Edward Hogan, Luigi Maccotta, Sophie Adler, Konrad Wagstyl

**Affiliations:** ^1^ UCL Great Ormond Street Institute of Child Health London UK; ^2^ School of Biomedical Engineering & Imaging Sciences King's College London London UK; ^3^ Neurobiology Research Unit Rigshospitalet Copenhagen Denmark; ^4^ Department of Neurology and Epileptology University of Tübingen Tübingen Germany; ^5^ Department of Neuroscience Monash University Melbourne Victoria Australia; ^6^ Department of Neurology Alfred Hospital Melbourne Victoria Australia; ^7^ Department of Neuroscience, School of Translational Medicine Monash University Melbourne Victoria Australia; ^8^ Amrita Advanced Center for Epilepsy Amrita Institute of Medical Sciences, Amrita Vishwa Vidyapeetham Kochi India; ^9^ Boston Children's Hospital Boston Massachusetts USA; ^10^ University of Iowa Health Care Iowa City Iowa USA; ^11^ Bristol Royal Hospital for Children Bristol UK; ^12^ Beijing Tiantan Hospital Capital Medical University Beijing China; ^13^ Central Emergency Military Hospital Bucarest Romania; ^14^ University Hospital of Wales Cardiff UK; ^15^ Department of Neurology, Welsh Epilepsy Centre University Hospital of Wales Cardiff UK; ^16^ Center for Neuroscience Research Children's National Research Institute, Children's National Hospital Washington District of Columbia USA; ^17^ Department of Neurology George Washington University School of Medicine Washington District of Columbia USA; ^18^ Department of Pediatrics George Washington University School of Medicine Washington District of Columbia USA; ^19^ CHU Sainte‐Justine Montreal Quebec Canada; ^20^ Université de Montréal Montreal Quebec Canada; ^21^ Collégial International Sainte‐Anne Lachine Quebec Canada; ^22^ Latin America Brain Health Institute Universidad Adolfo Ibanez Santiago Chile; ^23^ Department of Biophysics, School of Medicine Istanbul Medipol University Istanbul Türkiye; ^24^ School of Medicine Finis Terrae University Santiago Chile; ^25^ Biomedical Imaging Unit and Artificial Intelligence Foundation for the Promotion of Health and Biomedical Research of the Valencia Region ‐ Prince Felipe Research Center (FISABIO‐CIPF) València Spain; ^26^ Department of Neuroradiology University Hospital Freiburg Freiburg Germany; ^27^ Epilepsy Center, Medical Center–University of Freiburg, member of European Reference Network EpiCARE, Faculty of Medicine University of Freiburg Freiburg Germany; ^28^ Research Department Fundación Iniciativa Para las Neurociencias Madrid Spain; ^29^ Department of Neurology, Epilepsy Program Ruber International Hospital Madrid Spain; ^30^ Child Neuropsychiatry Unit, member of European Reference Network EpiCARE Istituto di Ricovero e Cura a Carattere Scientifico (IRCCS) Istituto Giannina Gaslini Genoa Italy; ^31^ Department of Neuroradiology IRCCS Istituto Giannina Gaslini Genoa Italy; ^32^ Division of Neurosurgery IRCCS Istituto Giannina Gaslini Genoa Italy; ^33^ DINOGMI, University of Genoa Genoa Italy; ^34^ IRCCS Istituto Giannina Gaslini Genoa Italy; ^35^ Department of Neurosciences, Rehabilitation, Ophthalmology, Genetics, Maternal and Child Health University of Genoa Genoa Italy; ^36^ Department of Neurosurgery Great Ormond Street Hospital London UK; ^37^ Radiology Department Great Ormond Street Hospital for Children London UK; ^38^ Hospital for Sick Children Toronto Ontario Canada; ^39^ Department of Neuroradiology Diagnostic Imaging Center Barcelona Spain; ^40^ Fundació de Recerca Clínic Barcelona Institut d'Investigacions Biomèdiques August Pi i Sunyer (FCRB‐IDIBAPS) Barcelona Spain; ^41^ Kuopio University Hospital, full member of European Reference Network EpiCARE Kuopio Finland; ^42^ University of Eastern Finland Kuopio Finland; ^43^ Neuroscience and Human Genetics Department Meyer Children's Hospital IRCCS Florence Italy; ^44^ University of Florence Florence Italy; ^45^ Department of Neurosurgery National Center of Neurology and Psychiatry Tokyo Japan; ^46^ Department of Radiology National Center of Neurology and Psychiatry Tokyo Japan; ^47^ Department of Psychiatry and Behavioral Science Juntendo University Graduate School of Medicine Tokyo Japan; ^48^ Department of Diagnostic Radiology Institute of Science Tokyo Japan; ^49^ CHU Lyon Lyon France; ^50^ Department of Neuroradiology Neurosurgery Institute Dr. A. Asenjo Santiago Chile; ^51^ Neurophysiology Unit Neurosurgery Institute Dr. A. Asenjo Santiago Chile; ^52^ Pediatric Neurosurgery Neurosurgery Institute Dr. A. Asenjo Santiago Chile; ^53^ Medical Physics Unit Bambino Gesù Children's Hospital, IRCCS Rome Italy; ^54^ Neurosurgery Unit Bambino Gesù Children's Hospital, IRCCS Rome Italy; ^55^ Neurology, Epilepsy, and Movement Disorders Bambino Gesù Children's Hospital, IRCCS Rome Italy; ^56^ Diagnostic and Interventional Neuroradiology Unit Bambino Gesù Children's Hospital, IRCCS Rome Italy; ^57^ Department of Radiology St. Luke's Hospital Thessaloniki Greece; ^58^ Epilepsy Center St. Luke's Hospital Thessaloniki Greece; ^59^ Birmingham Women's and Children's NHS Foundation Trust Birmingham UK; ^60^ Institute of Health and Neurodevelopment Aston University Birmingham UK; ^61^ UCL Queen Square Institute of Neurology London UK; ^62^ National Hospital for Neurology and Neurosurgery London UK; ^63^ University of Campinas Campinas Brazil; ^64^ Brazilian Institute of Neuroscience and Neurotechnology São Paulo Brazil; ^65^ Department of Neuropediatrics University Children's Hospital Zurich Zurich Switzerland; ^66^ MR‐Research Center University Children's Hospital Zurich Zurich Switzerland; ^67^ University of Zurich Zurich Switzerland; ^68^ Department of Neurology University of Pennsylvania Philadelphia Pennsylvania USA; ^69^ Department of Radiology University of Pennsylvania Philadelphia Pennsylvania USA; ^70^ Department of Biostatistics, Epidemiology, and Informatics University of Pennsylvania Philadelphia Pennsylvania USA; ^71^ Department of Neurology Washington University School of Medicine St. Louis Missouri USA; ^72^ Department of Radiology Washington University School of Medicine St. Louis Missouri USA

**Keywords:** artificial intelligence, epilepsy surgery, focal epilepsy, MRI, segmentation

## Abstract

**Objective:**

Quantitative assessment of extent of tissue resection following epilepsy surgery requires accurate delineation of the resection cavity on postoperative magnetic resonance imaging (MRI). Current methods for resection cavity masking are time‐consuming and labor‐intensive, and existing automated approaches exhibit variable segmentation accuracy, particularly on extratemporal resections. We developed MELD‐PostOp, a deep learning tool trained and evaluated on a large, heterogeneous cohort to automatically segment resection cavities.

**Methods:**

The study included 1.5‐ and 3T postoperative three‐dimensional T1‐weighted MRI images from the Multicentre Epilepsy Lesion Detection (MELD) project (*n*_subjects_ = 969, 27 centers) and from the EPISURG dataset (*n* = 133). The cohort included children and adults, alongside a range of resection locations, pathologies, and MRI characteristics. Resection cavities were individually segmented in 285 subjects and used to train an nnU‐Net prototype model. The prototype model was used to generate an additional 680 resection masks, which were subsequently quality‐controlled, edited, and combined with the original 285 to train the final MELD‐PostOp model (*n* = 965). A Stratified Test Cohort (*n* = 50) and Independent Test Cohort (*n* = 87) were withheld for model evaluation. Performance was evaluated using Dice similarity coefficient (DSC), 95th percentile Hausdorff distance (HD95), number of predicted clusters, and inference runtime, and compared against established tools (Epic‐CHOP, ResectVol, and RESSEG).

**Results:**

MELD‐PostOp achieved a median DSC of .85 and HD95 of 3.61 on the combined test cohort, outperforming Epic‐CHOP (DSC .69, HD95 9.67), ResectVol (DSC .66, HD95 15.05), and RESSEG (DSC .43, HD95 32.67), with significant improvements seen in both temporal and especially extratemporal resections. The model detected 98.5% (135/137) of resection cavities. MELD‐PostOp runtime was 17 s per MRI, compared to 612 s (ResectVol), 3205 s (Epic‐CHOP), and 4 s (RESSEG). MELD‐PostOp performance remained high across clinical and imaging subgroups (median DSC > .8).

**Significance:**

MELD‐PostOp is an open‐source research tool that provides an accurate, efficient, and generalizable solution for postoperative resection cavity segmentation using only postoperative MRI scans.

**Key points**

- MELD‐PostOp achieves high segmentation accuracy (DSC > .8), with consistently high‐quality segmentations across age groups, sex, image resolutions, and resection locations.
- The model was trained on 965 annotated T1‐weighted postoperative scans from sites worldwide, supporting its generalization across diverse clinical and imaging settings.
- The model automatically segments resection cavities from a postoperative T1 MRI scan in 17 s, a 36–188× faster runtime than existing SPM‐based tools.

## 1 INTRODUCTION

Epilepsy surgery is an established and effective treatment for patients with drug‐resistant focal epilepsy^1^ caused by structural etiologies, for which computational techniques may help to optimize resection strategies. Complete resection of the epileptogenic zone is necessary for achieving postoperative seizure freedom.^2^, ^3^ However, the resection extent must be balanced with limiting the removal of functional tissue to minimize neurocognitive consequences.^4^ For magnetic resonance imaging (MRI)‐visible lesions, postoperative MRI plays a key role in assessing resection extent and analyzing its association with postsurgical seizure freedom and neurocognitive outcomes. In patients undergoing a reoperation due to seizure recurrence, approximately 60% of cases were attributed to incomplete resection of the epileptogenic zone, often resulting from lesion mislocalization, residual epileptogenic tissue, or surgical limitations imposed by proximity to eloquent cortex.^5^ However, the evaluation of resection extent frequently relies on qualitative rather than quantitative assessments, that is, radiologists visually assess whether the lesion has been completely resected.^5^ Such visual assessments are subject to interrater variability, are dependent on reader expertise, and are usually binary (complete vs. incomplete), limiting the ability to assess whether the proportion of the structural lesion resected or resection of specific regions of the epileptogenic zone are important in determining postoperative outcomes. Accurate delineation of the resection cavity is essential for research into understanding and improving surgical outcomes.

Quantitative masks of resection extent can be generated from postoperative MRI scans with manual, semiautomated, and fully automated approaches.^6^, ^7^, ^8^, ^9^ Manual masking by experts remains the gold standard but is time‐consuming and varies between raters.^10^ Semiautomated methods accelerate this process by using computational algorithms to propagate user‐generated segmentation prompts into three‐dimensional (3D) masks. These are then manually reviewed and updated.^6^, ^7^ Although faster, even semiautomated segmentation of images limits the capacity to scale analysis to large cohorts with hundreds or thousands of subjects and is still subject to interrater variability.

Several studies have proposed automated approaches for resection cavity segmentation following epilepsy surgery.^8^, ^9^, ^11^, ^12^ These approaches mainly fall into two categories. The first involves coregistration of preoperative and postoperative scans, followed by voxel‐wise subtraction or intensity difference mapping.^8^, ^9^ Epic‐CHOP and ResectVol are two open‐source examples of this approach that are both built on the Statistical Parametric Mapping (SPM) toolbox. The second category encompasses deep learning (DL) methods, typically based on convolutional neural network (CNN) architectures,^13^ trained to segment the resection cavity using real or synthetic examples of postoperative scans with corresponding ground‐truth labels. RESSEG is one example of a DL approach trained using the open EPISURG dataset. EPISURG is an adult cohort of primarily temporal‐lobe resections.^11^, ^14^, ^15^

A number of limitations hamper the utility of both SPM‐based and DL methods. SPM‐based methods require accurate coregistration and tissue segmentation of pre‐ and postoperative images. This multistage process is time‐consuming and introduces potential sources of error. DL methods on the other hand are dependent on large sample sizes, which must accurately capture the true heterogeneity of the target population to generalize to independent external validation cohorts. For resection cavity masking, DL methods have, to date, only been trained on data from one or two centers. In empirical validation, existing SPM methods outperformed DL approaches. However, both methods failed to detect the resection on 12%–16% and 54%–56% of cases, respectively. Notably, all methods performed less well on extratemporal lesions, failing in 15%–30% (SPM‐based) and 85% (DL‐based) of cases.^16^ These limitations highlight the need for a robust, generalizable segmentation framework capable of accurately delineating resection cavities using only postoperative MRI data.

To address this, we collected a large, heterogeneous multicenter dataset of postoperative 3D T1‐weighted (T1w; 1.5 T or 3 T) MRI images from pediatric and adult patients with a range of epilepsy pathologies distributed across the brain. We introduce a scalable annotation strategy, combining manual labeling and pseudolabeling to define the resection cavities. We trained an nnU‐Net, a self‐configuring model that selects the optimal architecture and hyperparameters and has demonstrated state‐of‐the‐art performance across a range of biomedical segmentation tasks.^17^ We evaluated our model, MELD‐PostOp, on two withheld test cohorts and compared performance to existing approaches, Epic‐CHOP^9^, ResectVol,^8^ and RESSEG.^11^, ^14^, ^15^ MELD‐PostOp, a robust and generalizable model for segmentation of resection cavities on postoperative T1w scans, is provided open‐source for independent validation and research into optimizing surgical outcomes.

## 2 MATERIALS AND METHODS

### 2.1 Study population

#### 2.1.1 Datasets and inclusion criteria

This study used anonymized MRI data collected as part of the Multicentre Epilepsy Lesion Detection (MELD) Focal Epilepsies project (https://meldproject.github.io/), which has Health Research Authority approval (IRAS: 301863), and the open‐source EPISURG dataset. The MELD Focal Epilepsies dataset (v1.0, January 2025), collated from February 2022 to January 2025, includes 1090 participants with postoperative MRI scans from 27 epilepsy centers. Each participating center obtained local ethical approval to retrieve, anonymize, and share retrospective, routinely acquired clinical imaging data and clinical variables with the central MELD research team. Patients were included if they underwent lesionectomy or lobectomy for focal epilepsy and had a postoperative 3D T1w brain MRI scan. Excluded patients included those who had undergone disconnection procedures, laser ablation, or thermocoagulation.

Clinical variables included age, sex, pathology, surgical procedure and time from surgery to postoperative MRI. Age at surgery or age at preoperative scan, dependent on data availability, was classified as pediatric (<18 years) or adult (≥18 years). Pathologies were defined by postoperative histopathology or MRI diagnosis, dependent on data availability. These were grouped as (1) malformation of cortical development (MCD), including focal cortical dysplasia (FCD), periventricular nodular heterotopia, polymicrogyria, and mild malformation of cortical development with oligodendroglial hyperplasia and epilepsy; (2) cavernoma; (3) hippocampal sclerosis (HS); (4) low‐grade epilepsy‐associated tumor (LEAT); (5) dual pathology; (6) other; or (7) unknown. Dual pathology referred to cases in which more than one distinct pathological lesion was identified. “Other” pathologies included cortical gliosis, hypothalamic hamartoma, and normal MRI findings without a specific histopathological diagnosis. “Unknown” indicated cases where pathological information was unavailable or not specified in the anonymized clinical data received. Time from surgery to postoperative MRI was calculated based on the difference between year of surgery and year of postoperative scan. This was grouped into four groups: (1) surgery and postoperative scan occurring within the same calendar year, (2) postoperative scan performed in the year following surgery, (3) postoperative scan performed more than 2 years after surgery, and (4) unknown. The unknown category included cases in which year of surgery or year of postoperative scan were unavailable.

In addition, the open‐source EPISURG dataset (*n* = 430),^14^, ^15^ a clinical dataset of postoperative T1w MRI scans of adult epilepsy patients who underwent resective surgery, was incorporated. Available clinical variables included the surgical procedure and operated hemisphere. EPISURG patients were included if they had a 3D T1w postoperative scan with corresponding resection cavity masks.

#### 2.1.2 Cohort creation

A total of 1102 patients met the inclusion criteria: 969 patients from the MELD Focal Epilepsies dataset and 133 patients from the EPISURG dataset. These patients were organized into four cohorts: (1) Prototype Cohort (*n* = 285), (2) MELD‐PostOp Train Cohort (*n* = 965, including the Prototype Cohort), (3) Stratified Test Cohort (STC; *n* = 50), and (4) Independent Test Cohort (ITC; *n* = 87; Figure 2C). Figure 1 includes an overview of cohorts and how they were used to train and test the MELD‐PostOp model.

##### Prototype Cohort

To train the model, a fully quality‐controlled set of resection cavity masks for all 965 patients in the MELD‐PostOp Train Cohort needed to be created. To accelerate the manual process, a subset Prototype Cohort was semiautomatically segmented in a two‐stage process (*n* = 285). First, nnInteractive was used via the napari graphical user interface. This semiautomated tool uses a pretrained network to refine users' prompts to generate an initial delineation of resection cavities.^7^, ^18^ The masks were visually quality controlled and manually refined when necessary using ITK‐SNAP.^6^ Manual resection cavity masks for all 285 scans were generated by members of the team (*n* = 152, J.Se., R.P., B.H., E.N.) or downloaded as part of the EPISURG dataset (*n* = 133). Second, all resection cavity masks underwent subsequent visual quality control (QC) and, where necessary, correction by a single rater (J.Se.) prior to incorporation into the Prototype Cohort (Figure 1). No harmonization procedures were applied. These labeled images were used to train the Prototype nnU‐Net model (see Section 2.2 for nnU‐Net).

##### MELD‐PostOp Train Cohort

The MELD‐PostOp Train Cohort combines subjects in the Prototype Train Cohort (*n* = 285) with the remaining MELD Focal Epilepsies patients (*n* = 680). To generate labels for these 680 scans, the Prototype model was used to automatically predict resection cavities. Multiview QC images were reviewed for all 680 predicted cavities (Supplementary Figures S1 and S2).

Cases that failed QC (*n* = 86) were manually refined using ITK‐SNAP. The approved and manually edited segmentations were combined with the Prototype Train Cohort to form the MELD‐PostOp Train Cohort (*n* = 965). This MELD‐PostOp Train Cohort was used to train the final MELD‐PostOp model.

##### Stratified Test Cohort

To test differences in performance across clinical and demographic factors, 50 subjects from the full MELD Focal Epilepsies cohort were assigned to an STC that was withheld from all training and optimization experiments. Subjects were randomly selected, stratifying by site, age, and pathology.

##### Independent Test Cohort

To evaluate MELD‐PostOp performance on data from an unseen scanner, two MELD Focal Epilepsies sites were withheld as the ITC (total *n* = 87). Sites were chosen because they were large (*n* = 53; *n* = 34) with a range of pathologies and ages.

Ground‐truth segmentation masks for all subjects in both test cohorts were created with the same semiautomated segmentation workflow used for the Prototype Cohort.

##### Interrater variability in segmentation of resection cavities

To evaluate interrater variability in ground truth segmentation itself, 10 scans were randomly selected from the MELD sites used in the Prototype Cohort. Three independent raters including J.Se. performed segmentation under two conditions: segmentation using ITK‐SNAP only and using nnInteractive only. Resection cavity masks generated by J.Se. using ITK‐SNAP were part of ground truth masks. Interrater and intermethod agreement was quantified using the Dice similarity coefficient (DSC).

### 2.2 nnU‐Net resection cavity models

The Prototype and MELD‐PostOp models were trained using the nnU‐Net framework with the 3D full‐resolution configuration, with postoperative T1w 3D MRI scan as input, and resection cavity masks as the target segmentation. Fivefold cross‐validation was performed, with 80% of the data allocated for training and 20% for validation in each model. Folds were randomly stratified to maintain balanced label representation across the training and validation sets. The final model was an ensemble averaging predictions from the five models. All hyperparameters were automatically selected by nnU‐Net, except the number of epochs, which was increased to 2000.

### 2.3 Other automated segmentation tools

Three automated epilepsy surgery resection tools, Epic‐CHOP,^9^ ResectVol,^8^ and RESSEG,^11^, ^14^, ^15^ were selected as leading existing methods.^16^ Epic‐CHOP and ResectVol run within SPM12.^19^ Briefly, they automatically predict the resection cavity by coregistering the preoperative and postoperative scans and computing voxelwise differences. RESSEG runs on 3D U‐Net CNN, directly predicting resection cavity on postoperative scans. Methods are detailed in the original papers.^8^, ^9^, ^11^, ^14^, ^15^

Source code for each tool was downloaded from the following:

- Epic‐CHOP: https://github.com/iBrain‐Lab/EPIC‐CHOP
- ResectVol: https://www.lniunicamp.com/resectvol
- RESSEG: https://github.com/fepegar/resseg

**FIGURE 1:**
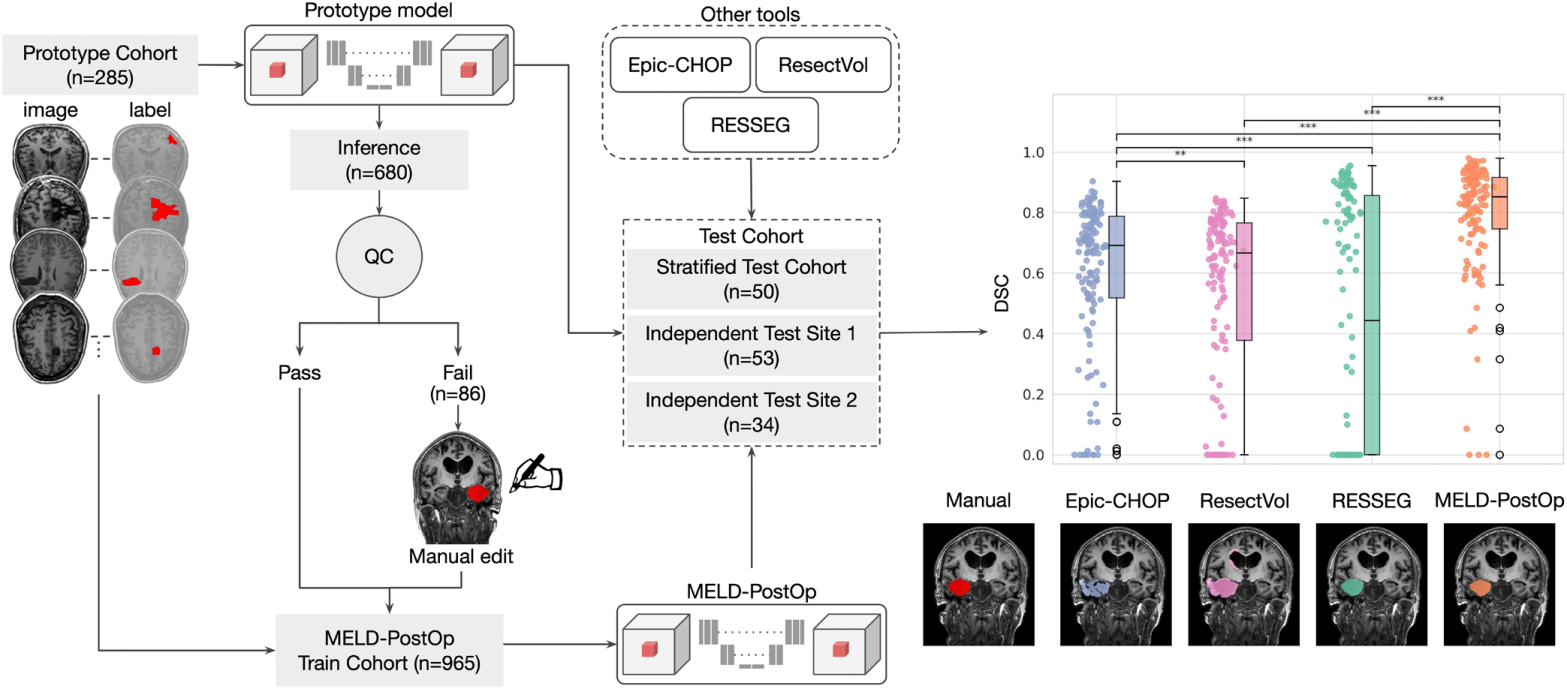
MELD‐PostOp overview. The Prototype Cohort (*n* = 285) was used to train the prototype model. The prototype model was applied to an inference cohort (*n* = 680). All predictions underwent visual quality control (QC). Failed cases were manually refined using nnInteractive and ITK‐SNAP. The masks that passed QC and manually edited masks were combined with the Prototype Cohort to form the MELD‐PostOp Train Cohort (*n* = 965), which was used to train the final MELD‐PostOp model. Model performance was evaluated on the test cohorts. MELD‐PostOp performance was compared against Epic‐CHOP, ResectVol, and RESSEG. DSC, Dice similarity coefficient. **p < .01, ***p < .001.

### 2.4 Evaluation of automated segmentation tools on test cohorts

MELD‐PostOp, Epic‐CHOP, ResectVol, and RESSEG were evaluated on the STC and ITC. Resection cavity masks were automatically segmented by each model. Evaluation metrics included the following: DSC and 95th percentile Hausdorff distance (HD95) between the ground truth mask and each automatically segmented mask, the number of predicted clusters, and model runtime.

DSC captures overall agreement between ground truth and predicted masks and was computed as: $\mathrm{DSC}=\frac{2\left(G\cap P\right)}{G+P}$, where *G* is the binary ground truth mask, and *P* is the binary automatically segmented masks. One limitation of DSC is that it tends to be lower for smaller objects. HD95 captures the distance between the boundaries of the objects and was computed as $\mathrm{HD}95=\max\left({\text{percentile}}_{95}\left(D\left(G\to P\right)\right),{\text{percentile}}_{95}\left(D\left(P\to G\right)\right)\right)$, where *D* is the shortest distance from the surface of one mask to the surface of the other. Model performance was summarized using the median DSC and HD95 with associated interquartile ranges (IQRs). The Wilcoxon signed‐rank test was applied to compare the median DSC between models. Also calculated were the proportions of cases with DSC > 0, that is, sensitivity of the model to detect a resection cavity, and DSC > .5, which represents the proportion of cases with a meaningful overlap.^16^

Clusters were defined as contiguous connected voxels, categorized as true positive (TP) if there was overlap with the ground truth mask and false positives (FP) if not. The median number of FP clusters per image with IQR is reported. Positive predictive value (PPV) was calculated as $\mathrm{PPV}=\frac{\mathrm{TP}}{\mathrm{TP}+\mathrm{FP}}$.

Mean runtime per image was measured by recording the runtime in seconds for each model applied to the same set of 10 postoperative images and calculating the mean and SD.

### 2.5 Subgroup analysis of model performance

Performance in the test cohorts (*n* = 137) was stratified by age group, sex, histopathology, resection lobe, surgical side, postoperative MRI field strength, isotropic image resolution, and time from surgery to postoperative MRI. Due to sample size limitations, for subgroups with more than two categories, the category with the highest median DSC was compared against all remaining categories (e.g., temporal vs. extratemporal) using Mann–Whitney *U*‐tests. Unknown categories were excluded from analysis. The relationship between DSC and resection volume was analyzed using Spearman rank correlation test.

Group‐stratified performance of MELD‐PostOp was also quantified combining the predictions on validation cohorts (i.e., cross‐validation predictions) with predictions on the test cohorts using pairwise *t*‐tests. Bonferroni adjustment was used to correct *p*‐values for multiple subgroup comparisons and were considered statistically significant if *p*_*adjusted*_ < .05.

To group scan acquisitions, MRI scans were considered isotropic if the resolution in all three dimensions was within ±.1, else anisotropic.

Postoperative images and their corresponding ground truth masks were nonlinearly registered to MNI‐ICBM152 space (Figure 2D).^20^, ^21^ Overlap with lobes from the Cerebrum Atlas (CerebrA)^22^ and the resection cavity masks was computed. The lobar label with the highest DSC was used to define the resection lobe and hemisphere. Cavities not overlapping a cortical lobe (e.g., hypothalamic hamartoma) were grouped into the “other” category. SynthSeg‐derived segmentations were used to QC coregistrations. Initial registrations were computed using ANTsPy.^23^ For those with mean DSC < .5, registrations were recomputed with EasyReg.^24^, ^25^

### 2.6 Code and model availability

All code for model design, development, and statistical analyses alongside the MELD‐PostOp model is available at the MELDProject Github.

## 3 RESULTS

### 3.1 Study population

#### 3.1.1 Patient characteristics

A total of 969 patients from 27 MELD sites and 133 patients from the EPISURG dataset were included in the study (total *n* = 1102). Demographic and clinical characteristics of the cohort are illustrated in Figure 2 and summarized in Table 1. The entire cohort comprised 45.0% pediatric and 54.7% adult patients, with 45.7% males and 42.2% females. Histopathological diagnoses were heterogeneous, with MCD being the most prevalent pathological group (38.2%), followed by LEAT (17.6%) and HS (17.2%). The temporal lobe was the most frequently resected (57.4%), followed by the frontal lobe (27.6%; Figure 2D). Resections were evenly distributed between left (51.5%) and right (48.5%) hemispheres. The majority of postoperative MRI scans were acquired at 3 T (76.7%) and were of isotropic resolution (72.6%). The majority of scans were obtained within the first postoperative year (median = 0 years [IQR = 0–1], range = 0–15 years).

**FIGURE 2:**
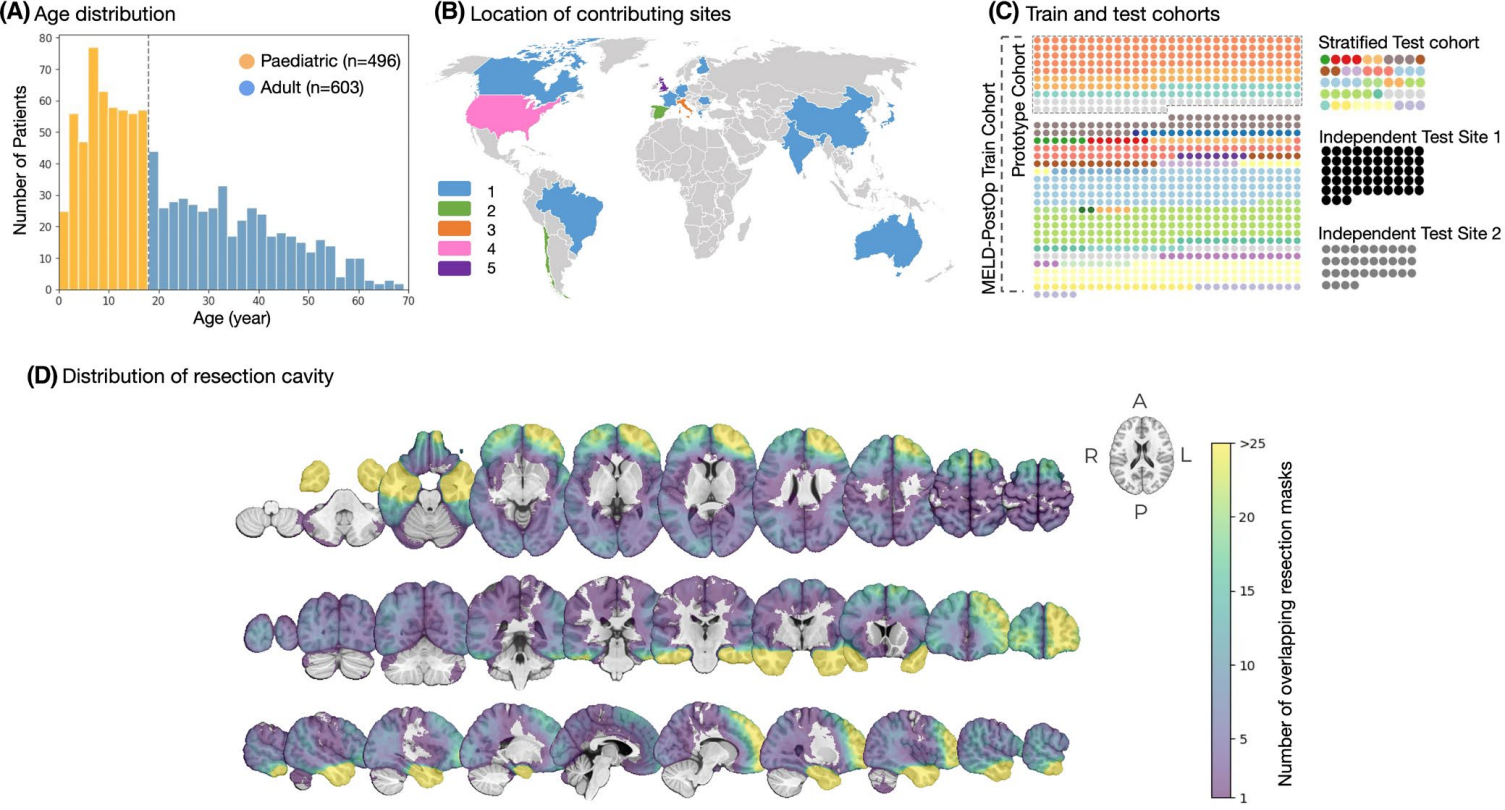
Visualization of patient characteristics in the study population. (A) Age distribution and classification of patients into pediatric (<18 years) and adult (≥18 years) groups. (B) Geographical distribution of contributing sites, with each color indicating the number of participating sites from a country. A total of 28 sites (MELD sites and EPISURG) across 17 countries contributed. (C) Cohort composition. Prototype Cohort (*n* = 285) included patients from three Multicentre Epilepsy Lesion Detection (MELD) sites and the EPISURG dataset. MELD‐PostOp Train Cohort (*n* = 965) incorporated Prototype Cohort and additional patients from 25 MELD sites. Randomly selected patients from 25 MELD sites formed the Stratified Test Cohort (*n* = 50), and two MELD sites served as the Independent Test Cohort (*n* = 53, *n* = 34). (D) Volumetric density map of manually segmented resection cavity masks in Montreal Neurological Institute space, demonstrating widespread coverage with the highest overlap density, that is, the most frequently operated locations, in the temporal and left frontal lobes. A, anterior; L, left; P, posterior; R, right.

**TABLE 1:** Clinical and imaging characteristics of the study population (*n* = 1102).

Characteristic	Number of patients	Percentage
Age		
Pediatric	496	45.0
Adult	603	54.7
Unknown	3	.3
Sex		
Male	504	45.7
Female	465	42.2
Unknown	133	12.1
Pathology		
MCD	421	38.2
HS	190	17.2
LEAT	194	17.6
Cavernoma	48	4.4
Dual pathology	83	7.5
Other	23	2.1
Unknown	143	13.0
Location of resection		
Temporal	633	57.4
Frontal	304	27.6
Occipital	39	3.5
Parietal	112	10.2
Insular	9	.8
Other	5	.5
Surgical side		
Left	567	51.5
Right	535	48.5
MRI field strength		
3 T	845	76.7
1.5 T	124	11.3
Unknown	133	12.1
Isotropy		
Isotropic	800	72.6
Anisotropic	302	27.4
Time from surgery to postoperative scan		
Same year	332	30.1
Following year	201	18.2
≥2 years	133	12.1
Unknown	436	39.6

Abbreviations: HS, hippocampal sclerosis; LEAT, low‐grade epilepsy‐associated tumor; MCD, malformation of cortical development; MRI, magnetic resonance imaging.

### 3.2 Overall model performance

Model performance on the STC and ITC across four models (MELD‐PostOp, Epic‐CHOP, ResectVol, and RESSEG) is summarized in Table 2 and illustrated in Figure 3. MELD‐PostOp achieved a median DSC of .85 across the two cohorts, significantly higher than Epic‐CHOP, ResectVol, and RESSEG (*p* < .001), and comparable to interrater and intermethod agreement (median DSC ≥ .82; Tables S4 and S5). There was a meaningful overlap (DSC > .5) in 93.4% of patients.

**TABLE 2:** Model performance of automated resection cavity segmentation models on the Stratified Test Cohort and Independent Test Cohort.

Model	Cohort	Median DSC [IQR]	DSC > 0 (%)	DSC > .5 (%)	Median HD95 [IQR]	PPV	Median FP clusters [IQR]	Mean runtime, s/image ± SD
Epic‐CHOP	Stratified	.67 [.51–.76]	41/50 (82.0)	34/50 (68.0)	10.84 [5.85–22.47]	.09	3 [1–12]	3205 ± 876
	Independent	.70 [.53–.80]	80/87 (92.0)	65/87 (74.7)	9.54 [6.67–15.22]	.25	3 [1–6]	
	Combined	.69 [.52–.79]	121/137 (88.3)	99/137 (72.3)	9.67 [5.94–16.85]	.17	11 [5–29]	
ResectVol	Stratified	.62 [.35–.73]	39/50 (78.0)	32/50 (64.0)	10.39 [6.48–32.93]	.35	1 [0–3]	612 ± 160
	Independent	.68 [.42–.76]	77/87 (88.5)	61/87 (70.1)	17.23 [10.46–33.42]	.20	1 [0–5]	
	Combined	.66 [.38–.76]	116/137 (84.7)	93/137 (67.9)	15.05 [8.47–33.72]	.24	1 [0–4]	
RESSEG	Stratified	.00 [.00–.80]	24/50 (48.0)	20/50 (42.0)	42.72 [8.99–90.10]	.50	.5 [0–1]	4 ± 1
	Independent	.67 [.00–.86]	51/87 (58.6)	47/87 (54.0)	29.32 [5.84–87.58]	.50	0 [0–1]	
	Combined	.43 [.00–.86]	75/137 (54.7)	67/137 (48.9)	32.67 [5.97–90.19]	.55	0 [0–1]	
MELD‐PostOp	Stratified	.83 [.69–.89]	50/50 (100)	46/50 (92.0)	3.87 [3.00–7.14]	.95	0 [0–0]	17 ± 5
	Independent	.87 [.76–.93]	85/87 (97.7)	82/87 (94.3)	3.32 [2.24–6.78]	.93	0 [0–0]	
	Combined	.85 [.74–.92]	135/137 (98.5)	128/137 (93.4)	3.61 [2.24–6.96]	.93	0 [0–0]	

*Note*: Performance metrics are presented as median DSC, DSC > 0, DSC > .5, median HD95, PPV, number of FP clusters, and mean runtime per image.

Abbreviations: DSC, Dice similarity coefficient; FP, false positive; HD95, 95th percentile Hausdorff distance; IQR, interquartile range; PPV, positive predictive value.

**FIGURE 3:**
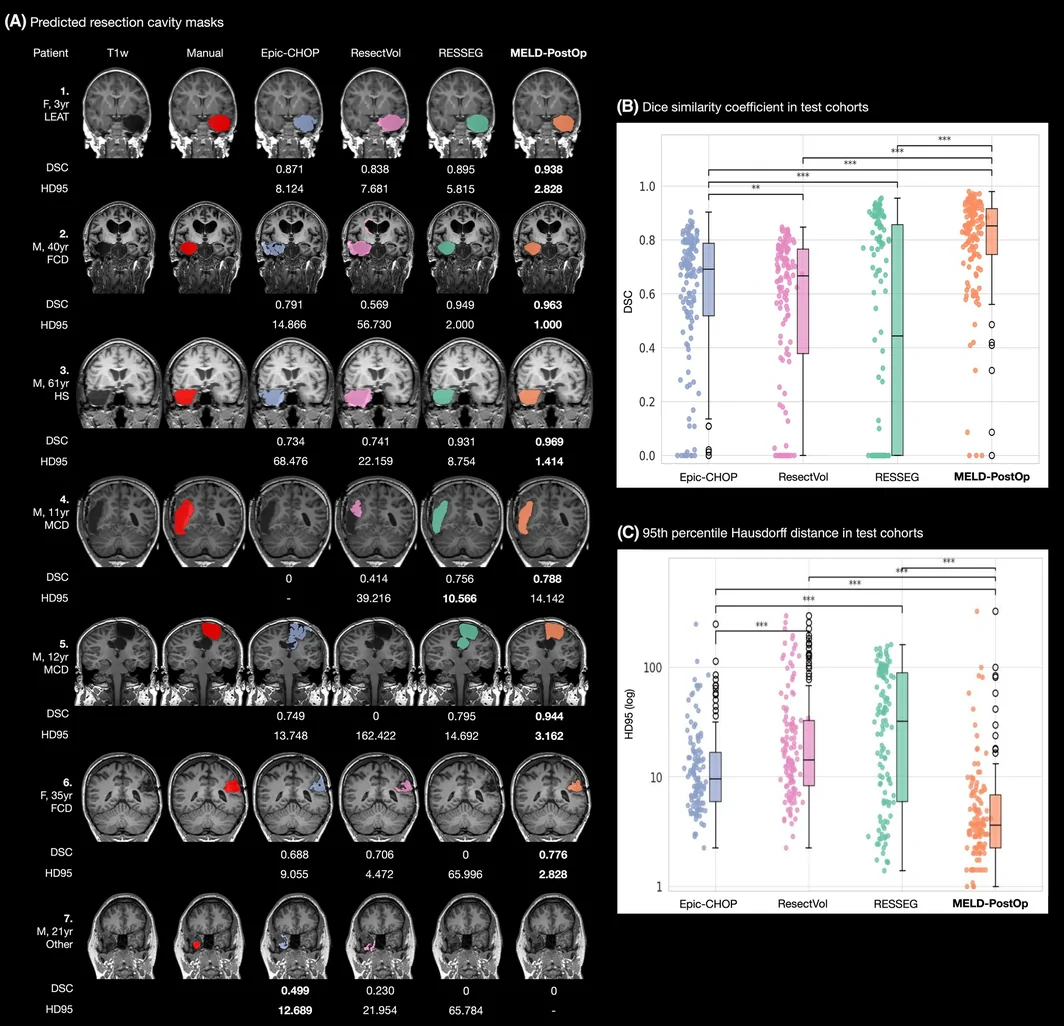
(A) Raw postoperative T1‐weighted (T1w) magnetic resonance imaging scans, ground truth manual resection masks, and automated segmentation from Epic‐CHOP, ResectVol, RESSEG, and MELD‐PostOp models. The number displayed beneath each predicted mask indicates the Dice similarity coefficient (DSC) and the 95th percentile Hausdorff distance (HD95) relative to the manual mask. (B, C) Box and scatter plots of DSC (B) and HD95 (C) across models in the combined test cohort (*n* = 137). MELD‐PostOp achieved a significantly higher median DSC and lower HD95 than Epic‐CHOP, ResectVol, and RESSEG (*p* < .001). F, female; FCD, focal cortical dysplasia; HS, hippocampal sclerosis; LEAT, low‐grade epilepsy‐associated tumor; M, male; MCD, malformations of cortical development. **p < .01, ***p < .001.

MELD‐PostOp successfully identified all resection cavities (DSC > 0) in the STC (100% sensitivity) and 85 of 87 resection cavities in the ITC (97.7% sensitivity). The two cases MELD‐PostOp failed to segment were in the smallest 6.6% by volume of resections (1.03 and 2.28 cm^3^). In comparison, Epic‐CHOP failed to segment a resection cavity in 16, ResectVol in 21, and RESSEG in 62 cases.

MELD‐PostOp had a significantly higher PPV  (.93, *p* < .001) compared to Epic‐CHOP (PPV = .17), ResectVol (PPV = .24), and RESSEG (PPV = .55), with FP predictions in only 6% across both test cohorts. Additionally, MELD‐PostOp had a median HD95 of 3.61, significantly lower than Epic‐CHOP (9.67), ResectVol (15.05), and RESSEG (32.67; *p* < .001).

Runtime analysis showed MELD‐PostOp had a mean processing time of 17 s per image (SD = 5 s), compared to 612 s (i.e., 10 min; SD = 160 s) for ResectVol and 3205 s (i.e., 53 min; SD = 876 s) for Epic‐CHOP (*p* < .001). RESSEG had the shortest mean processing time of 4 s per image (SD = 1 s).

Model outputs are visualized for a number of example cases (Figure 3A). The first three patients underwent temporal lobe resections for different temporal etiologies (LEAT, FCD, and HS). All models successfully segmented the temporal lobe resection cavities. However, MELD‐PostOp achieved higher DSC values exceeding .94 compared to less than .88 for Epic‐CHOP and ResectVol. The fourth to sixth examples represent patients with nontemporal resections due to underlying MCD and FCD. MELD‐PostOp accurately segmented these resection cavities, reaching DSCs of .79, .94, and .77, respectively. In contrast, Epic‐CHOP failed to produce a segmentation for example 4, and ResectVol failed on example 5. RESSEG failed to produce a segmentation for example 6 and exhibited ventricular oversegmentation in example 5. The last example represents one of the two failure cases of MELD‐PostOp, involving a small temporal lobe resection.

### 3.3 Subgroup analysis of model performance

Comparing model performance in subgroups (Tables 3 and S3), MELD‐PostOp achieved significantly higher segmentation performance in both temporal and extratemporal resections compared with Epic‐CHOP, ResectVol, and RESSEG. Across all four models, DSC was significantly higher in temporal than in extratemporal cases. MELD‐PostOp exhibited the smallest reduction in performance between temporal and extratemporal resections (median DSC difference = .07) compared to Epic‐CHOP, ResectVol, and RESSEG (median DSC difference = .13, .18, and .73, respectively). MELD‐PostOp also exhibited higher DSC on HS than other pathologies. A significant positive correlation was observed in the combined test cohort between the volume of the ground truth resection masks and the DSC (*r* = .56, corrected *p* < .001), meaning that larger resections had better automated delineation of the cavity. It is worth noting that the DSC is biased against smaller masks, where a single voxel error will have a greater impact. Nevertheless, across the smallest 10% of cavities, the median DSC was still .87 (IQR = .54–.94). Furthermore, no significant correlation was observed between the volume of ground truth cavities and the HD95 metric. MELD‐PostOp achieved a consistent median HD95 < 4.1 and showed no significant difference between subgroups. Separate subgroup analyses for STC and ITC are presented in Tables S6 and S7.

**TABLE 3:** Model performance breakdown across clinical and imaging variables (*n* = 137).

Variable	Epic‐CHOP		ResectVol		RESSEG		MELD‐PostOp	
	DSC	HD95	DSC	HD95	DSC	HD95	DSC	HD95
Age								
Pediatric	.68 [.41–.79]	13.90 [8.22–21.76]	.61 [.00–.74]	22.95 [7.60–56.58]	.10 [.00–.80]	50.97 [12.17–93.36]	.84 [.78–.91]	3.61 [2.24–7.62]
Adults	.69 [.53–.79]	8.25 [5.80–14.73]	.68 [.43–.77]	14.30 [9.12–30.33]	.63 [.00–.86]	31.76 [5.58–84.81]	.86 [.74–.92]	3.61 [2.24–6.73]
Sex								
Male	.70 [.51–.79]	11.12 [5.83–18.32]	.62 [.37–.76]	19.10 [10.40–35.27]	.32 [.00–.85]	37.22 [8.51–98.73]	.85 [.73–.91]	3.61 [2.24–7.21]
Female	.68 [.56–.77]	8.28 [6.89–14.22]	.70 [.47–.78]	10.74 [7.60–26.57]	.53 [.00–.86]	24.54 [5.68–81.56]	.87 [.78–.93]	3.61 [2.45–6.73]
Pathology								
HS	.73 [.67–.80]	7.21 [5.39–24.92]	.76 [.71–.78]*	12.57 [9.05–22.33]	.86 [.77–.93]**	8.51 [3.01–17.99]	.91 [.85–.94]*	3.16 [2.00–4.69]
Non‐HS	.68 [.51–.78]	9.70 [6.60–16.72]	.62 [.35–.74]*	16.03 [8.13–39.43]	.05 [.00–.81]**	41.36 [9.27–94.31]	.83 [.70–.91]*	3.61 [2.29–7.33]
Location of resection								
Temporal	.71 [.59–.80]*	8.06 [5.75–12.81]	.70 [.57–.77]**	12.88 [8.47–25.69]	.73 [.00–.89]*	27.66 [4.32–54.61]	.87 [.80–.93]*	3.32 [2.24–5.15]
Extratemporal	.58 [.29–.72]*	14.05 [8.12–21.40]	.52 [.00–.70]**	24.22 [8.89–70.72]	.00 [.00–.76]*	81.56 [10.57–110.48]	.80 [.67–.90]*	3.93 [2.73–8.12]
Surgical side								
Left	.63 [.50–.75]	8.25 [5.93–15.97]	.66 [.23–.76]	14.70 [7.38–38.06]	.73 [.00–.89]	27.66 [4.32–54.61]	.85 [.69–.91]	3.67 [2.45–6.91]
Right	.70 [.55–.80]	10.68 [6.56–17.06]	.66 [.54–.76]	15.39 [9.25–31.75]	.00 [.00–.76]	81.56 [10.57–110.48]	.86 [.77–.92]	3.61 [2.24–6.61]
MRI field strength								
3 T	.70 [.53–.79]	9.43 [5.92–15.84]	.66 [.38–.77]	14.58 [8.16–34.10]	.55 [.00–.86]	31.69 [5.84–90.35]	.85 [.74–.92]	3.61 [2.24–7.14]
1.5 T	.56 [.11–.64]	17.03 [8.45–24.39]	.58 [.47–.69]	19.30 [11.00–28.31]	.00 [.00–.00]	50.84 [40.49–88.05]	.82 [.78–.89]	4.03 [3.00–5.38]
Isotropy								
Isotropic	.69 [.52–.79]	9.67 [6.11–16.85]	.65 [.37–.76]	16.40 [9.17–34.37]	.43 [.00–.85]	37.22 [5.99–90.35]	.85 [.73–.91]	3.61 [2.45–7.28]
Anisotropic	.72 [.63–.77]	10.32 [5.18–18.09]	.73 [.69–.75]	7.81 [6.16–11.58]	.46 [.00–.88]	18.66 [5.07–63.00]	.86 [.83–.93]	3.22 [1.80–3.90]
Time from surgery to postoperative scan								
Same year	.70 [.50–.79]	10.69 [6.28–18.74]	.68 [.37–.77]	13.98 [7.78–39.32]	.39 [.00–.78]	35.08 [9.43–90.27]	.84 [.72–.91]	3.80 [2.83–9.12]
≥1 year	.69 [.52–.77]	9.87 [6.89–15.21]	.68 [.38–.77]	16.40 [8.68–31.57]	.61 [.00–.88]	32.02 [5.83–88.80]	.86 [.76–.92]	3.32 [2.24–5.00]
Mask volume								
Coefficient, *r*_*s*_	.48**	−.02	.32***	.06	.66***	−.58***	.56***	−.15

*Note*: Median DSC [IQR] and median HD95 [IQR] for Epic‐CHOP, ResectVol, RESSEG, and MELD‐PostOp models are categorized by patient age, sex, pathology, resection location, surgical laterality, postoperative MRI field strength, isotropy, and time from surgery to postoperative scan.

Abbreviations: DSC, Dice similarity coefficient; HD95, 95th percentile Hausdorff distance; HS, hippocampal sclerosis; MRI, magnetic resonance imaging.

* *p* < .05;

** *p*< 0.01;

*** *p* < 0.001.

Analysis of the combined test cohort was underpowered to identify significant associations in certain stratified analyses (Table S8). Therefore, performance breakdown was additionally conducted on the entire cohort, combining validation predictions and the test cohorts, as presented in Tables S9 and S10. MELD‐PostOp model achieved a consistent median DSC > .80 across all subgroups except Other Resection (DSC = .61). Median DSCs were comparable between pediatric and adult patients, and between males and females, with no statistically significant differences (corrected *p* > .05). Performance varied by pathology, with the highest median DSC observed in HS (DSC = .98) significantly better than for MCD, LEAT, cavernoma, and unknown cases (corrected *p* < .05–.001). Median DSC remained high across all resection locations, with the temporal lobe achieving the highest performance (DSC = .96), followed by the frontal (DSC = .93) and occipital lobes (DSC = .93). Temporal lobe resection showed significantly higher DSC than frontal and parietal resection (corrected *p* < .05). No significant difference was observed between isotropic and anisotropic scans or in time from surgery to postoperative scan.

### 3.4 Interrater and intermethod variability in segmentation of resection cavities

Interrater and intermethod agreement for segmentation variability was quantified using the DSC. Across raters, median DSC values were consistently ≥ .82. Intermethod agreement between two expert‐guided segmentation methods (ITK‐SNAP and nnInteractive) demonstrated median DSC > .82 for all three raters. A mixed effect linear regression model showed no significant rater effect (Rater 1 vs. Rater 3: *β* = .002, *p* = .895; Rater 2 vs. Rater 3: *β* = −.021, *p* = .167). Against the MELD‐PostOp predictions, ITK‐SNAP‐delineated cavities had slightly higher DSC values than for nnInteractive (*β* = −.028, *p* = .022; Tables S4 and S5, Figure S11). MELD‐PostOp scores on the test cohorts are therefore not likely to be inflated by the use of nnInteractive.

Within the Prototype Cohort, two datasets, MELD (*n* = 152) and EPISURG (*n* = 133), were included. To assess potential systematic differences between the two sources, model performance was compared across datasets. Median DSCs were .88 and .90 for MELD and EPISURG, respectively, representing a small but statistically significant difference (*p* < .02; Figure S4).

## 4 DISCUSSION

We present MELD‐PostOp, an automated, deep learning‐based research tool designed to enable fast and accurate resection cavity segmentation from postoperative MRI scans in epilepsy patients. MELD‐PostOp significantly outperformed two state‐of‐the‐art automated resection cavity masking tools on independent test cohorts, with increased segmentation accuracy, fewer failures, and faster evaluation. MELD‐PostOp was trained and tested on a large and heterogeneous international multicenter dataset, and overall performance remained high across MRI acquisition parameters and demographics, although some variability was observed between subgroups. Median DSC for MELD‐PostOp was .85, consistent with interrater agreement scores, suggesting that the model approached the upper limit of achievable agreement given inherent variability in expert manual segmentation. MELD‐PostOp is a robust, open‐source tool that can be used by the field to pursue a range of research questions that aim to understand neuroimaging predictors of seizure freedom and neurocognitive outcomes to ultimately help improve epilepsy surgery outcomes.

### 4.1 Heterogeneous multicenter data for model development and evaluation

MELD‐PostOp has been trained and tested on >1000 postoperative MRI scans from epilepsy centers worldwide. Using a combined test cohort of 137 patients, our MELD‐PostOp model significantly outperformed current leading methods, which rely on registration and intensity comparisons with the preoperative MRI scan.^8^, ^9^ Methods reliant on differences in intensity between scans exhibit a number of limitations. First, other preoperative to postoperative brain changes, such as changes in ventricle size and postoperative brain shift, may lead to intensity changes and result in inaccurate segmentation of nonresected areas. An example of this is in the segmentation of Patient 5's postoperative resection cavity by Epic‐CHOP (Figure 3). Second, these algorithms depend on tissue segmentation differences, and missegmentation of preoperative lesional tissue, for example, due to cystic changes being misattributed as cerebrospinal fluid, can ultimately lead to undersegmentation of the resection cavity. Third, any misregistration of the postoperative MRI to the preoperative MRI will impact the subsequent segmentation. This is a particular challenge in patients with large resections or young children, where there may be significant brain growth and development between the pre‐ and postoperative MRI. Although these SPM‐based intensity models have previously been shown to outperform DL approaches on smaller datasets,^16^ the MELD Focal Epilepsies large dataset size and diverse resection locations, combined with the state‐of‐the‐art nnUNet architecture, likely underpin the significantly improved performance, particularly on extratemporal resections. The inclusion of pediatric cohorts is particularly valuable, as these patients often present with different imaging characteristics, lesion locations, and histologies from adults. With the increasing move toward earlier surgery, quantitative postoperative analyses of these cohorts with respect to surgical seizure freedom and neurocognitive outcomes are essential. Moreover, multicenter datasets enable the capture of rarer pathologies and diverse imaging sequences within the training set, thereby enhancing model robustness and generalizability.

### 4.2 Accelerating costly labeling of large datasets through pseudolabeling

A major challenge in developing robust segmentation models is the need for large, accurately annotated datasets, which are costly and time‐consuming to produce and require expertise. In this study, we adopted a pseudolabeling strategy to mitigate this challenge. A prototype model was initially trained on 285 MRI scans with manually annotated masks, and its predictions were quality‐controlled and then used to generate labels for an additional 680 scans. This iterative approach substantially reduced the annotation burden while enabling the creation of a large training set. Such strategies demonstrate how pseudolabeling can be used in neuroimaging research to accelerate dataset expansion and improve model performance without proportionally increasing expert labeling effort.

### 4.3 Open science

By making MELD‐PostOp openly available, we aim to provide the community with a robust tool for postoperative cavity segmentation that can be readily integrated into research workflows. Open‐source availability not only facilitates independent replication but also enables benchmarking against existing models, fostering quantitative comparison. Automated and reliable postoperative resection cavity tools combined with large multicenter datasets have the potential to enable big data analysis of neurosurgical outcomes in epilepsy. MELD‐PostOp provides a fast, feasible solution for automated masking of large datasets.

### 4.4 Enabling quantitative analysis of postsurgical outcomes

The ability to rapidly, reliably segment large numbers of postsurgical resection cavities could prove invaluable for advancing our understanding of predictors of surgical outcomes. Evidence from recent studies highlights several promising neuroimaging predictors of seizure freedom that need further validation and exploration. For instance, within FCD type II lesions, ultra‐high‐resolution 7 T imaging revealed a “black line sign,” complete resection of which was predictive of postoperative seizure freedom.^26^ Other studies have demonstrated that disconnection of thalamocortical white matter tracts in frontal lobe lesions^27^ or diffusion‐derived gray and white matter abnormalities in temporal lobe epilepsy^28^ were associated with improved seizure freedom. Resection of the temporal piriform cortex in patients with temporal lobe epilepsy, and particularly in HS, has been linked to increased rates of seizure freedom in both adults^29^, ^30^ and children.^31^ Beyond seizure outcomes, Spyrantis et al. explored the relationship between the extent of resection and psychobehavioral outcomes in temporal lobe epilepsy. They found that volume of resection was significantly correlated with divided attention, quality of life, and depressive symptoms, whereas sparing of mesial temporal lobe structures for neocortical lesions was associated with fewer depressive symptoms and improved quality of life.^4^ Collectively, these studies indicate that our understanding of which lesional features and anatomical structures require resection to reliably achieve seizure freedom remains incomplete. Furthering our understanding could lead to more individualized targeting of procedures that reduce the risk of adverse neurocognitive outcomes. Comprehensive analysis of surgical procedures, pre and post‐surgical imaging and postsurgical outcomes in large cohorts will be a key to understanding this complexity,^32^, ^33^, ^34^, ^35^ for which accurate and robust automated computational tools such as MELD‐PostOp are essential.

### 4.5 Limitations

This study has a number of limitations. First, MELD‐PostOp has been trained and tested on only patients who underwent resective surgical procedures, specifically lesionectomies, and lobectomies and not interventions such as laser interstitial thermal therapy or disconnection surgeries.^36^, ^37^ Independent datasets and models will be required to segment the area of ablated or disconnected tissue. Second, MELD‐PostOp has not been evaluated on intraoperative MRI data or MRI data from the initial postoperative period, where inflammation, bleeding, and swelling may impact its performance. Use on such scans will require careful validation and likely model retraining on representative data. Third, contrast status was unavailable for most MRI scans (Table S11). Consequently, the dataset may have included a mixture of pre‐ and postcontrast T1w images, and the impact of contrast enhancement on segmentation performance could not be assessed. In addition, information on whether scans were obtained following an initial surgery or a reoperation was not available in the dataset. Reoperation cases may present more complex radiological cases, which could further impact both ground truth delineation and automated segmentation performance. Fourth, Epic‐CHOP and ResectVol were designed to define preoperative tissue resected, rather than resection cavities, which could contribute to lower DSCs. However, the significantly reduced failure rate (DSC > 0) in MELD‐PostOp compared to all other models indicates there is also a considerable underlying performance gap. Finally, although MELD‐PostOp was evaluated on two independent test sites, both belong to the MELD consortium. As a result, truly independent external validation by researchers outside the consortium has not yet been performed. To facilitate external validation, we have made the code publicly available.

## 5 CONCLUSIONS

MELD‐PostOp is an automated research tool for segmenting resection cavities from postoperative MRI scans in epilepsy patients. It has improved performance and rapid inference time compared to existing models. The model's robustness to heterogeneous and unseen data, combined with its minimal input requirements, underscores its suitability for research applications. MELD‐PostOp is provided as a fully automated and open‐source pipeline to enable external validation as well as large‐scale quantitative analysis of postoperative imaging.

## AUTHOR CONTRIBUTIONS

**Jieun Seo:** Software; formal analysis; methodology; validation; visualization; writing—original draft. **Mathilde Ripart, Cornelius Kronlage, and Helene Kaas:** Formal analysis; validation. **Sophie Adler and Konrad Wagstyl:** Conceptualization; funding acquisition; supervision; writing—review and editing. **Ben Sinclair, Lucy Vivash, Merran R. Courtney, Terence J. O'Brien, Siby Gopinath, Harilal Parasuram, Sedat Kandemirli, Natally Alarab, Lillian Lai, Marcus Likeman, Kai Zhang, Jiajie Mo, Georgian Ciobotaru, James Galea, Philip Sequeiros‐Peggs, Khalid Hamandi, Hua Xie, Venkata Sita Priyanka Illapani, William D. Gaillard, Nathan T. Cohen, Alexander G. Weil, Florence Henrichon‐Goulet, Kenza S. Lahlou, Aristides Hadjinicolaou, Agustín Ibáñez, Gonzalo M. Rojas‐Costa, Horst Urbach, Lara Bücheler, Marcel Heers, Adrián Valls Carbó, Rafael Toledano, Giulia Nobile, Costanza Parodi, Domenico Tortora, Aswin Chari, Antonella Riva, Mariasavina Severino, Martin Tisdall, Felice D'Arco, Kshitij Mankad, Alessandro Consales, Maria H. Eriksson, Rory J. Piper, J. Helen Cross, Torsten Baldeweg, Sofia González‐Ortiz, Jose Pariente, Nuria Bargalló, Yawu Liu, Reetta Kälviäinen, Carmen Barba, Matteo Lenge, Renzo Guerrini, Masaki Iwasaki, Daichi Sone, Hiroyuki Maki, Tomoki Imokawa, Noriko Sato, Julien Jung, Francisco Sepulveda, Daniel Mansilla, Andres Goycoolea, Ingeborg Lopez, Antonio Napolitano, Alessandro De Benedictis, Luca De Palma, Maria Camilla Rossi‐Espagnet, Nikolaos Kondylidis, Kostakis Gkiatis, Kyriakos Garganis, Joshua Pepper, Stefano Seri, John S. Duncan, Clarissa L. Yasuda, Lucas Scárdua‐Silva, Marina K. M. Alvim, Fernando Cendes, Antonio G. Gennari, Ruth O'Gorman Tuura, Georgia Ramantani, Mariam Josyula, Joel Stein, Nishant Sinha, Kate Davis, R. Edward Hogan, and Luigi Maccotta:** Resources; data curation; writing—review and editing.

## FUNDING INFORMATION

J.Se., M.R., and K.W. are supported by Wellcome Trust (301991/Z/23/Z) and Epilepsy Research Institute UK (P2208). S.A. is supported by Epilepsy Research Institute UK (P2208). T.J.O. is supported by NHMRC Investigator Grants (APP1176426 & APP2034258). C.K. is supported by the DFG and the Faculty of Medicine Tübingen (MINT‐CS), the Else Kroener Fresenius Stiftung (PRECISE.net), and the Michael Foundation. H.X. is supported by the National Institute of Child Health and Human Development (P50HD105328‐01). N.T.C. is supported by the National Institute of Neurological Disorders and Stroke and the National Institutes of Health (K23NS131522). A.G.W. and A.H. are supported by the Canadian Institute for Health Research, Fonds de recherche du Québec–Santé, Savoy Foundation, Department of Surgery at Université de Montréal, and CHU Sainte‐Justine Foundation. A.I. is supported by grants from the Fogarty International Center, National Institutes of Health, National Institutes of Aging (R01 AG057234, R01 AG075775, R01 AG21051, R01 AG083799, CARDS‐NIH, R01 AG057234), Alzheimer's Association (SG‐20‐725707), Rainwater Charitable Foundation, ANID/FONDECYT Regular (1250091, 1210176 and 1220995), ANID/PIA/ANILLOS ACT210096, JPI JPND‐Care, DISCeRN 2025, FONDEF ID20I10152, ANID/FONDAP 15150012, Wellcome Trust (BRAIN‐CLIMA 335293/Z/25/Z), and CliCBrain (Horizon ID: 101236426; DOI 10.3030/101236426, Marie Skłodowska‐Curie Actions). A.Ch. is supported by NIHR & GOSH BRC. M.H.E. is supported by the Sigrid Jusélius Foundation. R.J.P. is supported by NIHR & GOSH Children's Charity. J.S.D. is supported by NIHR. C.L.Y. is supported by CNPQ (445340/2024‐0/; 313263/2025‐6). F.C. is supported by Sao Paulo Research Foundation (FAPESP) grants 2013/07559‐3 and 2021/12956‐8. The content of this article is solely the responsibility of the authors and does not necessarily represent the official views of any of the funders. The funders had no role in study design, data collection and analysis, decision to publish, or preparation of the manuscript.

## CONFLICT OF INTEREST STATEMENT

A.G.W. is a consultant for Monteris. K.D. is an advisory board member for NeuroPace, Rapport Therapeutics, Mosaica Therapeutics, and UCB. All other authors have no conflicts of interest to disclose.

## ETHICS STATEMENT

We confirm that we have read the Journal's position on issues involved in ethical publication and affirm that this report is consistent with those guidelines.

## Supporting information


**Table S1.** Pathological epileptogenic lesion types in the Multicentre Epilepsy Lesion Detection Focal Epilepsy dataset were determined using histopathological diagnosis. Where histopathological diagnosis was not available, radiological diagnosis was used. For analysis, pathologies were grouped into pathology categories listed below.
**Table S2.** Location of resection was determined using CerebrA atlas. Ground truth masks were nonlinearly registered to MNI‐ICBM152, and overlap between the registered masks and CerebrA labels was computed. Atlas labels were grouped into lobes as listed below, and masks not overlapping a cortical lobe were grouped into the “other” category.
**Table S3.** Demographic and clinical characteristics of patients from Stratified Test Cohort (*n* = 50), Independent Test Cohort (*n* = 87), and Combined Test Cohort (total *n* = 137).
**Table S4‐1.** Interrater variability analysis. Three independent raters segmented 10 resection cavities using ITK‐SNAP and nnInteractive separately. Pairwise agreement between raters is reported as mean Dice similarity coefficient (DSC) and median DSC. Median DSCs were consistently ≥.82 across all comparisons. Higher interrater agreement was observed for segmentation performed with nnInteractive compared with ITK‐SNAP.
**Table S4‐2.** Intermethod variability analysis comparing segmentations generated by ITK‐SNAP and nnInteractive for three independent raters. Agreement between methods is reported as mean Dice similarity coefficient (DSC) and median DSC. Median DSCs were ≥.82 across raters.
**Table S5.** Intermethod variability analysis comparing segmentations to MELD‐PostOp. Dice similarity coefficient (DSC) was computed between cross‐validation predictions and segmentations generated with two methods by three independent raters. A mixed effect linear regression model, using ITK‐SNAP segmentations from rater 3 as the reference condition, showed no significant rater effect (Rater 1 vs. Rater 3: *p* = .895, Rater 2 vs. Rater 3: *p* = .167). Segmentation methods demonstrated a small but significant effect (*p* = .022), with nnInteractive producing slightly lower DSC values compared with ITK‐SNAP.
**Table S6.** Performance breakdown by model across clinical and imaging variables in the Stratified Test Cohort (*n* = 50). Median Dice similarity coefficient [interquartile range (IQR)] and median 95th percentile Hausdorff distance [IQR] for Epic‐CHOP, ResectVol, RESSEG, and MELD‐PostOp models are categorized by patient age, sex, pathology, resection location, surgical laterality, postoperative magnetic resonance imaging (MRI) field strength, isotropy, and time from surgery to postoperative MRI.
**Table S7.** Performance breakdown by model across clinical and imaging variables in the Independent Test Cohort (*n* = 87). Median Dice similarity coefficient [interquartile range (IQR)] and median 95th percentile Hausdorff distance [IQR] for Epic‐CHOP, ResectVol, RESSEG, and MELD‐PostOp models are categorized by patient age, sex, pathology, resection location, surgical laterality, postoperative magnetic resonance imaging (MRI) field strength, isotropy, and time from surgery to postoperative MRI.
**Table S8.** Subgroup comparisons of MELD‐PostOp performance in the combined test cohort (*n* = 137) using the pairwise *t*‐test. Multiple comparison correction was applied using Bonferroni adjustment for subgroup level analysis. Categories containing fewer than two observations were excluded from the analysis (excluded categories: insular and other resection locations).
**Table S9.** Subgroup comparisons of MELD‐PostOp performance across the total cohort (*n* = 1102) using the pairwise *t*‐test. Multiple comparison correction was applied using Bonferroni adjustment for subgroup level analysis.
**Table S10.** MELD‐PostOp model subgroup performance across clinical and imaging variables evaluated on the entire cohort (*n* = 1102).
**Table S11.** Summary of magnetic resonance imaging acquisition characteristics of postoperative T1‐weighted scans from the Multicentre Epilepsy Lesion Detection dataset. Reported parameters include sequence family, contrast status, and slice thickness.
**Figure S1.** Representative 3*14 visual quality control (QC) image from a case that passed QC.
**Figure S2.** Representative 3*14 visual quality control (QC) image from a case that failed QC. The resection cavity segmentation was incomplete and subsequently corrected through manual editing.
**Figure S3.** Examples of resection segmentations that failed quality control. Segmentations were classified as failed if they met one or more of the following criteria: (1) incorrect anatomical location (e.g., off‐brain or unrelated region), (2) incomplete coverage of the resection cavity, or (3) inclusion of substantial noncavity tissue.
**Figure S4.** Raincloud plot showing the distribution of Dice similarity coefficient (DSC) for the Prototype Cohort from the Multicentre Epilepsy Lesion Detection (MELD; *n* = 152) and EPISURG (*n* = 133) datasets. The median DSC was .88 for MELD and .90 for EPISURG. A Mann–Whitney *U*‐test demonstrated a statistically significant difference (*p* = .011), with effect size of .157.
**Figure S5.** Raincloud plot illustrating change in Dice similarity coefficient (DSC) for cases that failed quality control after initial inference with the Prototype model (*n* = 86), comparing Prototype and MELD‐PostOp performance. Paired values depict within‐case changes in segmentation accuracy. Overall DSC increased with MELD‐PostOp, indicating improved segmentation performance. Median DSC increased from .02 (interquartile range [IQR] = .00–.91) for the Prototype model to .65 (IQR = .19–.90) for MELD‐PostOp. This difference was significant (*p* < .01).
**Figure S6.** Spearman rank correlation between manual resection cavity volume and Dice similarity coefficient (DSC) for Combined Test Cohort (*n* = 137), Stratified Test Cohort (*n* = 50), and Independent Test Cohort (*n* = 87).
**Figure S7.** Spearman rank correlation between manual resection cavity volume and 95th percentile Hausdorff distance for Combined Test Cohort (*n* = 137), Stratified Test Cohort (*n* = 50), and Independent Test Cohort (*n* = 87).
**Figure S8.** One of two failure cases of MELD‐PostOp. This patient was an adult male with cavernoma. Ground truth volume is 1030 mm^3^. MELD‐PostOp failed to identify the resection cavity, generating no predicted clusters. Epic‐CHOP and RESSEG produced false positive segmentations, whereas ResectVol generated a true positive cluster (Dice similarity coefficient = .354). The second failure case is presented in Figure 3A (Patient 7).
**Figure S9.** Example of false positive case. The left panel shows the ground truth mask in a three‐dimensional (3D) view, and in two different slices from a single patient. The right panel shows the MELD‐PostOp predictions for the same patient, also in 3D view and in two corresponding slices. MELD‐PostOp predicted two clusters: one true positive and one false positive, resulting in a positive predictive value of .5.
**Figure S10.** Runtime analysis of Epic‐CHOP, ResectVol, RESSEG, and MELD‐PostOp was performed on the same 10 subjects using identical computational hardware. Epic‐CHOP demonstrated a median runtime of 3363.5 s, ResectVol 659.0 s, RESSEG 4.0 s, and MELD‐PostOp 15.0 s. Significant differences were observed between all four models (*p* < .001). Mean runtime was 3205 s, 612 s, 4.2 s, and 17 s, respectively.
**Figure S11.** Bland–Altman analysis comparing Dice similarity coefficient (DSC) between MELD‐PostOp cross‐validation predictions and nnInteractive, and ITK‐SNAP segmentations across 40 cases. The central dashed line indicates the mean DSC difference (mean = −.025), and the dotted lines represent the 95% limits of agreement (−.160 to .109). Thirty‐seven of 40 subjects fell within the limits of agreement, with three outliers, indicating overall good agreement between the two segmentation approaches.

## Data Availability

All code for model design, development, and statistical analyses alongside the MELD‐PostOp model is available at the MELDProject Github (https://github.com/MELDProject/MELD‐PostOp). Postoperative resection cavities can be requested from the MELD project. Postoperative MRI data for individual participants from the MELD Focal Epilepsies dataset cannot be shared due to ethical restrictions. The EPISURG dataset is available at https://rdr.ucl.ac.uk/articles/dataset/EPISURG_a_dataset_of_postoperative_magnetic_resonance_images_MRI_for_quantitative_analysis_of_resection_neurosurgery_for_refractory_epilepsy/9996158.
