# Supplementary material for: Automated segmentation of postsurgical resection cavities on magnetic resonance imaging in focal epilepsy: A Multicentre Epilepsy Lesion Detection study
Source: Epilepsia. 2026 Aug 19;67(11):5901–18. doi: 10.1002/epi.70450 (PMC13768940; doi:10.1002/epi.70450)

Supplementary Materials

Automated Segmentation of Post-Surgical Resection Cavities on MRI in Focal Epilepsy : A MELD Study

---

Supplementary Tables

Supplementary Table 1.
Pathological epileptogenic lesion types in the Multicentre Epilepsy Lesion Detection (MELD) Focal Epilepsy (FE) dataset were determined using histolopathological diagnosis. Where histopathological diagnosis was not available, radiological diagnosis was used. For analysis, pathologies were grouped into pathology categories listed below.

(1) MCD
Focal cortical dysplasia type I (FCD I)
Focal cortical dysplasia type IIa (FCD IIa)
Focal cortical dysplasia type IIb (FCD IIb)
Focal cortical dysplasia type II not otherwise specified (FCD II other)
Focal cortical dysplasia type IIIb (FCD IIIb)
Focal cortical dysplasia not otherwise specified (FCD other)
Malformation of cortical development (MCD)
Mild malformation of cortical development (mMCD)
Mild malformation of cortical development with oligodendroglial hyperplasia and epilepsy (MOGHE)
Polymicrogyria
Periventricular nodular heterotopia (PNH)
**(2) HS**
Hippocampal sclerosis (HS) / Mesial temporal sclerosis (MTS)
Hippocampal gliosis only
**(3) LEAT**
Dysembryoplastic neuroepithelial tumour (DNET)
Ganglioglioma
Astrocytoma
Polymorphic low-grade neuroepithelial tumour of the young (PLNTY)
Other low-grade tumour
Low-grade epilepsy-associated neuroepithelial tumour not otherwise specified (LEAT other)
**(4) Cavernoma**
Cavernoma
**(5) Dual pathology**
Dual pathology
**(6) Other pathology**
Cortical gliosis only
Hypothalamic hamartoma (HH)
Other pathology
Normal
**(7) Unknown**
Unknown / Missing data

Supplementary Table 2.

Location of resection was determined using CerebrA atlas. Ground truth masks were non-linearly registered to MNI-ICBM152 and overlap between the registered masks and CerebrA labels were computed. Atlas labels were grouped into lobes as listed below, and masks not overlapping a cortical lobe were grouped into the 'other' category.

(1) Temporal
Fusiform
Inferior temporal / Superior temporal
Middle temporal
Transverse temporal
Entorhinal
Parahippocampal / Hippocampus
Amygdala
Isthmus cingulate
**(2) Frontal**
Caudal middle frontal / Rostral middle frontal
Lateral orbitofrontal / Medial orbitofrontal
Pars opercularis / Pars orbitalis / Pars triangularis
Paracentral / Precentral
Superior frontal
Caudal anterior cingulate / Rostral anterior cingulate
**(3) Occipital**
Ceneus
Lateral occipital
Lingual
Pericalcarine
**(4) Parietal**
Inferior parietal / Superior parietal
Postcentral
Precuneus
Supramarginal
posterior cingulate
**(5) Insular**
Insula
**(6) Other**
Ventricles / Ventral Diencephalon / Cerebellum GM,WM / Vermal lobules I-X
Thalamus / Caudate / Putamen / Pallidum
Brainstem / Accumbens area / Basal forebrain / Optic chiasm

Supplementary Table 3.
Demographic and clinical characteristics of patients from Stratified Test Cohort (n=50), Independent Test Cohort (n=87), and Combined Test Cohort (total n=137).

	Stratified Test Cohort (n=50)		Independent Test Cohort (n=87)		Combined Test Cohort (n=137)	
	**Number of patients (n)**	**Percentage (%)**	**Number of patients (n)**	**Percentage (%)**	**Number of patients (n)**	**Percentage (%)**
Age						
Paediatric	18	36	15	17.2	33	24.1
Adult	30	60	72	82.8	102	74.5
Unknown	2	4	0	0.0	2	1.5
Sex						
Male	31	62	62	71.3	93	67.9
Female	19	38	25	28.7	44	32.1
Pathology						
MCD	19	38	37	42.5	56	40.9
HS	9	18	12	13.8	21	15.3
LEAT	9	18	15	17.2	24	17.5
Cavernoma	2	4	8	9.2	10	7.3
Dual pathology	9	18	5	5.7	14	10.2
Other	2	4	8	9.2	10	7.3
Unknown	0	0	2	2.3	2	1.5
Location of resection						
Temporal	33	66	51	58.6	84	61.3
Frontal	9	18	23	26.4	32	23.4
Occipital	3	6	2	2.3	5	3.6
Parietal	4	8	10	11.5	14	10.2
Insular	1	2	0	0.0	1	0.7
Other	0	0	1	1.1	1	0.7
Surgical side						
Left	22	44	43	49.4	65	47.4
Right	28	56	44	50.6	72	52.6
MRI field strength						
3T	42	84	85	97.7	127	92.7
1.5T	8	16	2	2.3	10	7.3
Isotropy						
Isotropic	42	84	85	97.7	127	92.7
Anisotropic	8	16	2	2.3	10	7.3
Time from surgery to postoperative scan						
Same year	15	30	25	28.7	40	29.2
Following year	12	24	31	35.6	43	31.4
≥ 2 years	4	8	22	25.3	26	19.0
Unknown	19	38	9	10.3	28	20.4

MCD, malformation of cortical development; HS, hippocampal sclerosis; LEAT, low grade epilepsy associated tumour; DSC, dice similarity coefficient; IQR, interquartile range

Supplementary Table 4-1.

Inter-rater variability analysis. Three independent raters segmented 10 resection cavities using ITK-SNAP and nnInteractive separately. Pairwise agreement between raters is reported as mean DSC and median DSC. Median DSCs were consistently ≥ 0.82 across all comparisons. Higher inter-rater agreement was observed for segmentation performed with nnInteractive compared with ITK-SNAP.

Method	Comparison	Mean DSC ± SD	Median DSC [IQR]
ITK-SNAP	Rater 1 – Rater 2	0.85 ± 0.06	0.85 [0.81-0.89]
	Rater 1 – Rater 3	0.82 ± 0.08	0.84 [0.73-0.88]
	Rater 2 – Rater 3	0.81 ± 0.09	0.82 [0.76-0.86]
nnInteractive	Rater 1 – Rater 2	0.91 ± 0.06	0.93 [0.86-0.94]
	Rater 1 – Rater 3	0.90 ±0.04	0.89 [0.87-0.93]
	Rater 2 – Rater 3	0.88 ± 0.07	0.91 [0.88-0.92]

Supplementary Table 4-2.

Inter-method variability analysis comparing segmentations generated by ITK-SNAP and nnInteractive for three independent raters. Agreement between methods is reported as mean DSC and median DSC. Median DSCs were ≥ 0.82 across raters.

Rater	Mean DSC ± SD	Median DSC [IQR]
Rater 1	0.90 ± 0.04	0.91 [0.88-0.93]
Rater 2	0.80 ± 0.09	0.82 [0.75-0.87]
Rater 3	0.83 ± 0.07	0.85 [0.75-0.89]

Supplementary Table 5.

Inter-method variability analysis comparing segmentations to MELD-PostOp. DSC was computed between cross-validation predictions and segmentations generated with 2 methods by 3 independent raters. A mixed effect linear regression model, using ITK-SNAP segmentations from rater 3 as the reference condition, showed no significant rater effect (Rater 1 vs Rater 3: $p = 0.895$, Rater 2 vs Rater 3: $p = 0.167$). Segmentation methods demonstrated a small but significant effect ($p = 0.022$), with nnInteractive producing slightly lower DSC values compared with ITK-SNAP.

Method	Comparison	Mean DSC ± SD	Median DSC [IQR]
ITK-SNAP	Rater 1 – MELD-PostOp	0.82 ± 0.08	0.82 [0.77-0.87]
	Rater 2 – MELD-PostOp	0.81 ± 0.10	0.81 [0.77-0.89]
	Rater 3 – MELD-PostOp	0.84 ± 0.12	0.88 [0.84-0.91]
nnInteractive	Rater 1 – MELD-PostOp	0.82 ± 0.09	0.84 [0.77-0.88]
	Rater 2 – MELD-PostOp	0.78 ± 0.14	0.82 [0.72-0.89]
	Rater 3 – MELD-PostOp	0.79 ± 0.12	0.83 [0.78-0.85]

Supplementary Table 6.

Performance breakdown by model across clinical and imaging variables in the STC (n=50). Median DSC [IQR] and median HD95 [IQR] for Epic-CHOP, ResectVol, RESSEG, and MELD-PostOp models categorised by patient age, sex, pathology, resection location, surgical laterality, postoperative MRI field strength, isotropy and time from surgery to postoperative MRI.

	Epic-CHOP		ResectVol		RESSEG		MELD-PostOP	
	**DSC**	**HD95**	**DSC**	**HD95**	**DSC**	**HD95**	**DSC**	**HD95**
Age								
Paediatric	0.63 [0.34-0.77]	14.25 [6.57-22.47]	0.60 [0.00-0.68]	13.93 [7.00-35.57]	0.00 [0.00-0.61]	55.20 [15.40-101.56]	0.83 [0.63-0.85]	3.87 [2.59-7.29]
Adults	0.67 [0.57-0.76]	8.25 [5.84-18.56]	0.65 [0.40-0.78]	9.83 [6.08-37.58]	0.43 [0.00-0.89]	35.07 [3.99-83.58]	0.86 [0.70-0.89]	3.61 [3.00-5.86]
Sex								
Male	0.61 [0.50-0.77]	11.51 [5.92-22.47]	0.62 [0.35-0.72]	15.85 [6.93-31.62]	0.10 [0.00-0.78]	38.32 [10.47-96.29]	0.83 [0.67-0.87]	4.12 [3.08-7.61]
Female	0.67 [0.56-0.75]	8.28 [5.29-22.90]	0.65 [0.34-0.78]	9.22 [5.51-39.69]	0.00 [0.00-0.84]	54.02 [7.69-86.60]	0.85 [0.79-0.89]	3.61 [2.34-5.55]
Pathology								
HS	0.71 [0.59-0.76]	6.93 [5.95-24.92]	0.73 [0.64-0.81]	9.27 [5.58-20.38]	0.85 [0.00-0.89]	9.64 [3.93-45.30]	0.86 [0.83-0.91]	4.12 [3.16-4.90]
Non-HS	0.65 [0.49-0.76]	11.45 [5.83-21.40]	0.60 [0.14-0.73]	10.70 [6.65-41.53]	0.00 [0.00-0.77]	50.97 [15.01-92.22]	0.83 [0.63-0.88]	3.74 [3.00-7.62]
Location of resection								
Temporal	0.67 [0.56-0.76]	8.28 [5.85-16.39]	0.64 [0.49-0.77]	9.27 [6.16-27.46]	0.32 [0.00-0.88]	31.82 [4.12-56.39]	0.85 [0.77-0.88]	4.00 [3.00-6.08]
Extra-temporal	0.62 [0.08-0.75]	16.82 [7.83-32.70]	0.45 [0.00-0.67]	20.17 [7.05-108.80]	0.00 [0.00-0.13]	90.51 [27.68-104.29]	0.80 [0.59-0.91]	3.74 [3.00-8.12]
Surgical side								
Left	0.65 [0.55-0.73]	7.03 [5.85-22.30]	0.65 [0.36-0.77]	9.41 [5.83-31.42]	0.00 [0.00-0.77]	56.39 [8.23-95.82]	0.84 [0.70-0.87]	4.85 [3.39-7.76]
Right	0.67 [0.51-0.77]	11.81 [6.26-21.76]	0.60 [0.31-0.72]	10.39 [7.00-44.87]	0.20 [0.00-0.81]	35.14 [9.42-89.28]	0.83 [0.68-0.89]	3.31 [2.00-5.40]
MRI field strength								
3T	0.67 [0.54-0.77]	10.69 [5.83-21.40]	0.62 [0.35-0.75]	9.27 [6.32-32.93]	0.20 [0.00-0.83]	35.07 [6.67-90.08]	0.84 [0.65-0.89]	3.87 [3.00-7.14]
1.5T	0.56 [0.05-0.64]	17.03 [6.56-24.92]	0.58 [0.34-0.68]	16.86 [9.82-43.23]	0.00 [0.00-0.00]	71.00 [44.01-92.40]	0.82 [0.76-0.88]	4.03 [3.00-5.78]
Isotropy								
Isotropic	0.64 [0.52-0.76]	10.84 [5.94-23.35]	0.60 [0.09-0.73]	12.46 [6.48-41.53]	0.00 [0.00-0.77]	45.92 [12.88-90.10]	0.83 [0.65-0.88]	4.41 [3.00-7.56]
Anisotropic	0.76 [0.51-0.80]	8.48 [4.78-13.74]	0.73 [0.69-0.78]	7.81 [6.62-10.42]	0.46 [0.00-0.86]	18.66 [7.54-63.57]	0.86 [0.83-0.94]	2.80 [1.65-4.17]
Time from surgery to postoperative scan								
Same year	0.69 [0.62-0.76]	11.45 [6.56-22.83]	0.62 [0.09-0.72]	14.00 [6.58-63.91]	0.32 [0.00-0.79]	38.46 [9.07-88.06]	0.82 [0.66-0.90]	3.16 [3.00-7.42]
≥ 1 year	0.58 [0.43-0.72]	10.69 [6.44-16.80]	0.68 [0.37-0.77]	7.07 [5.99-18.35]	0.05 [0.00-0.88]	38.56 [4.07-85.45]	0.84 [0.63-0.86]	3.93 [3.53-5.75]
Mask volume								
Coefficient (r_s)	0.36	-0.02	0.32	-0.08	0.52 ***	-0.4 *	0.41 *	0.04

*, p <0.05; **, p< 0.01; ***, p<0.001; ns, not significant; HD95, 95th percentile Hausdorff distance; HS, hippocampal sclerosis; MRI, magnetic resonance imaging

Supplementary Table 7.

Performance breakdown by model across clinical and imaging variables in the ITC (n=87). Median DSC [IQR] and median HD95 [IQR] for Epic-CHOP, ResectVol, RESSEG, and MELD-PostOp models categorised by patient age, sex, pathology, resection location, surgical laterality, postoperative MRI field strength, isotropy and time from surgery to postoperative MRI.

	**Epic-CHOP**		**ResectVol**		**RESSEG**		**MELD-PostOP**	
	**DSC**	**HD95**	**DSC**	**HD95**	**DSC**	**HD95**	**DSC**	**HD95**
Age								
Paediatric	0.71 [0.50-0.84]	13.10 [8.54-17.12]	0.62 [0.11-0.78]	30.68 [10.55-70.39]	0.46 [0.00-0.90]	29.53 [8.19-85.27]	0.89 [0.81-0.94]	3.16 [2.24-6.06]
Adults	0.70 [0.53-0.79]	8.56 [5.81-14.66]	0.69 [0.45-0.76]	16.22 [10.48-30.27]	0.68 [0.00-0.86]	28.80 [5.84-86.73]	0.86 [0.75-0.93]	3.46 [2.24-6.76]
Sex								
Male	0.71 [0.52-0.80]	10.37 [5.77-16.95]	0.66 [0.40-0.76]	20.81 [11.43-38.00]	0.63 [0.00-0.89]	34.95 [7.55-98.34]	0.86 [0.75-0.93]	3.24 [2.24-6.09]
Female	0.69 [0.58-0.80]	8.62 [7.19-11.19]	0.70 [0.54-0.77]	13.93 [9.17-26.32]	0.77 [0.00-0.86]	17.23 [5.50-48.83]	0.90 [0.78-0.93]	3.61 [2.45-6.78]
Pathology								
HS	0.76 [0.72-0.80]	7.54 [5.15-15.26]	0.76 [0.73-0.77]	15.72 [10.48-22.24]	0.88 [0.80-0.93] **	7.87 [2.93-16.91]	0.92 [0.89-0.94]	2.83 [1.65-3.74]
Non-HS	0.69 [0.51-0.79]	9.67 [6.73-15.22]	0.66 [0.39-0.76]	17.23 [10.90-39.43]	0.43 [0.00-0.84] **	38.90 [6.38-97.95]	0.85 [0.74-0.92]	3.61 [2.24-7.14]
Location of resection								
Temporal	0.73 [0.65-0.80] *	7.87 [5.74-12.69]	0.71 [0.60-0.77] *	14.46 [10.46-22.33]	0.79 [0.00-0.89]	24.60 [5.10-50.31]	0.91 [0.81-0.94]	3.00 [2.00-4.48]
Extra-temporal	0.58 [0.36-0.71] *	13.75 [8.12-17.55]	0.52 [0.22-0.71] *	25.81 [10.81-41.11]	0.00 [0.00-0.79]	66.72 [9.82-118.10]	0.80 [0.73-0.90]	4.12 [2.64-7.70]
Surgical side								
Left	0.60 [0.50-0.75]	9.64 [6.71-15.30]	0.67 [0.23-0.74]	17.23 [12.08-41.45]	0.00 [0.00-0.88]	64.78 [6.30-105.14]	0.85 [0.71-0.93]	3.16 [2.24-4.50]
Right	0.71 [0.67-0.81]	9.43 [6.65-14.87]	0.68 [0.58-0.77]	17.61 [10.44-31.22]	0.69 [0.00-0.85]	25.83 [5.22-44.29]	0.88 [0.78-0.93]	3.61 [2.24-7.74]
MRI field strength								
3T	0.70 [0.52-0.80]	9.24 [6.53-15.07]	0.68 [0.41-0.77]	16.40 [10.44-34.01]	0.69 [0.00-0.87]	29.32 [5.83-90.19]	0.87 [0.76-0.93]	3.32 [2.24-6.96]
1.5T	0.59 [0.56-0.61]	17.10 [13.72-20.48]	0.61 [0.57-0.66]	21.01 [19.94-22.08]	0.32 [0.16-0.48]	27.24 [20.09-34.40]	0.86 [0.84-0.88]	4.24 [3.62-4.86]
Isotropy								
Isotropic	0.70 [0.52-0.80]	9.54 [6.53-15.07]	0.67 [0.41-0.77]	17.23 [10.49-32.83]	0.67 [0.00-0.86]	29.32 [5.84-90.19]	0.87 [0.76-0.93]	3.32 [2.24-6.96]
Anisotropic	0.70 [0.70-0.71]	19.02 [14.04-24.01]	0.70 [0.70-0.70]	19.24 [11.86-26.63]	0.45 [0.22-0.67]	34.50 [18.75-50.25]	0.85 [0.81-0.88]	3.22 [3.02-3.41]
Time from surgery to postoperative scan								
Same year	0.70 [0.49-0.81]	10.19 [5.44-16.62]	0.70 [0.44-0.78]	13.96 [8.83-34.01]	0.46 [0.00-0.77]	24.60 [10.57-90.19]	0.87 [0.74-0.92]	4.90 [2.83-8.88]
≥ 1 year	0.70 [0.57-0.79]	9.70 [7.00-15.00]	0.68 [0.40-0.74]	18.87 [10.44-32.83]	0.67 [0.00-0.86]	32.02 [5.84-92.03]	0.88 [0.77-0.94]	3.00 [2.24-4.14]
Mask volume								
Coefficient (r_s)	0.52 ***	0.01	0.31 *	0.14	0.72 ***	-0.66 ***	0.65 ***	-0.274

*, p <0.05; **, p< 0.01; ***, p<0.001; ns, not significant; HD95, 95th percentile Hausdorff distance; HS, hippocampal sclerosis; MRI, magnetic resonance imaging

Supplementary Table 8.

Subgroup comparisons of MELD-PostOp performance in the combined test cohort (n=137) using the pairwise t-test. Multiple comparison correction was applied using Bonferroni adjustment for subgroup level analysis. Categories containing fewer than two observations were excluded from the analysis (excluded categories: insular and other resection locations).

Comparison	t	p	Sig.	Corrected p	Sig. Corr.
Paediatric-Adult	-0.505	6.16E-01	ns	1.00E+00	ns
Paediatric-Unknown	-0.762	5.10E-01	ns	1.00E+00	ns
Adult-Unknown	-0.509	6.87E-01	ns	1.00E+00	ns
Male-Female	-0.941	3.49E-01	ns	1.00E+00	ns
Male-Unknown	-0.525	6.00E-01	ns	1.00E+00	ns
Female-Unknown	1.204	2.29E-01	ns	1.00E+00	ns
MCD-HS	-3.344	1.34E-03	**	5.91E-02	ns
MCD-LEAT	0.776	4.43E-01	ns	1.00E+00	ns
MCD-Cavernoma	1.925	8.45E-02	ns	1.00E+00	ns
MCD-Dual pathology	-0.141	8.90E-01	ns	1.00E+00	ns
MCD-Other	1.234	2.46E-01	ns	1.00E+00	ns
MCD-Unknown	0.321	8.01E-01	ns	1.00E+00	ns
HS-LEAT	2.559	1.61E-02	*	1.00E+00	ns
HS-Cavernoma	2.676	2.45E-02	*	1.00E+00	ns
HS-Dual pathology	1.433	1.72E-01	ns	1.00E+00	ns
HS-Other	2.064	6.75E-02	ns	1.00E+00	ns
HS-Unknown	0.862	5.45E-01	ns	1.00E+00	ns
LEAT-Cavernoma	1.5	1.60E-01	ns	1.00E+00	ns
LEAT-Dual pathology	-0.673	5.06E-01	ns	1.00E+00	ns
LEAT-Other	0.804	4.36E-01	ns	1.00E+00	ns
LEAT-Unknown	0.074	9.51E-01	ns	1.00E+00	ns
Cavernoma-Dual pathology	-1.842	8.89E-02	ns	1.00E+00	ns
Cavernoma-Other	-0.613	5.48E-01	ns	1.00E+00	ns
Cavernoma-Unknown	-0.886	4.60E-01	ns	1.00E+00	ns
Dual pathology-Other	1.19	2.54E-01	ns	1.00E+00	ns
Dual pathology-Unknown	0.354	7.74E-01	ns	1.00E+00	ns
Other-Unknown	-0.416	7.18E-01	ns	1.00E+00	ns
Temporal-Frontal	1.575	1.22E-01	ns	1.00E+00	ns
Temporal-Occipital	0.461	6.66E-01	ns	1.00E+00	ns
Temporal-Parietal	1.235	2.36E-01	ns	1.00E+00	ns
Frontal-Occipital	-0.402	7.00E-01	ns	1.00E+00	ns
Frontal-Parietal	0.168	8.68E-01	ns	1.00E+00	ns
Occipital-Parietal	0.476	6.44E-01	ns	1.00E+00	ns
Left-Right	-1.232	2.20E-01	ns	1.00E+00	ns
3T-1.5T	-0.512	6.16E-01	ns	1.00E+00	ns
3T-Unknown	0.816	4.15E-01	ns	1.00E+00	ns
1.5T-Unknown	-1.109	2.69E-01	ns	1.00E+00	ns
Isotropy-Anisotropy	-1.589	1.34E-01	ns	1.00E+00	ns
Same year-Following year	0.315	7.53E-01	ns	1.00E+00	ns
Same year-≥2 years	-1.756	8.41E-02	ns	1.00E+00	ns
Same year-Unknown	-0.1	9.21E-01	ns	1.00E+00	ns
Following year-≥2years	-1.8	7.70E-02	ns	1.00E+00	ns
Following year-Unknown	-0.393	6.96E-01	ns	1.00E+00	ns
≥2 years-Unknown	1.54	1.31E-01	ns	1.00E+00	ns

Sig., significance; Sig. corr., significance after bonferroni adjustment; LH, left hemisphere; RH, right hemisphere; MCD, malformation of cortical development; HS, hippocampal sclerosis; LEAT, low grade epilepsy associated tumour; *, p <0.05; **, P< 0.01; ***, p<0.001; ns, not significant.

Supplementary Table 9.
Subgroup comparisons of MELD-PostOp performance across the total cohort (n=1102) using the pairwise t-test. Multiple comparison correction was applied using Bonferroni adjustment for subgroup level analysis.

Comparison	t	p	Sig.	Corrected p	Sig. Corr.
Paediatric-Adult	0.064	9.49E-01	ns	1.00E+00	ns
Paediatric-Unknown	0.073	9.48E-01	ns	1.00E+00	ns
Adult-Unknown	0.062	9.56E-01	ns	1.00E+00	ns
Male-Female	-1.567	1.17E-01	ns	1.00E+00	ns
Male-Unknown	-0.525	6.00E-01	ns	1.00E+00	ns
Female-Unknown	1.204	2.29E-01	ns	1.00E+00	ns
MCD-HS	-5.355	1.28E-07	***	6.78E-06	***
MCD-LEAT	-0.335	7.38E-01	ns	1.00E+00	ns
MCD-Cavernoma	2.66	1.05E-02	*	5.54E-01	ns
MCD-Dual pathology	-0.162	8.71E-01	ns	1.00E+00	ns
MCD-Other	1.427	1.67E-01	ns	1.00E+00	ns
MCD-Unknown	-0.252	8.01E-01	ns	1.00E+00	ns
HS-LEAT	3.901	1.16E-04	***	6.14E-03	*
HS-Cavernoma	4.01	2.03E-04	***	1.07E-02	*
HS-Dual pathology	2.702	7.99E-03	**	4.24E-01	ns
HS-Other	2.499	2.01E-02	*	1.00E+00	ns
HS-Unknown	5.603	4.45E-08	***	2.36E-06	***
LEAT-Cavernoma	2.717	8.84E-03	**	4.69E-01	ns
LEAT-Dual pathology	0.06	9.52E-01	ns	1.00E+00	ns
LEAT-Other	1.495	1.48E-01	ns	1.00E+00	ns
LEAT-Unknown	0.16	8.73E-01	ns	1.00E+00	ns
Cavernoma-Dual pathology	-2.544	1.33E-02	*	7.06E-01	ns
Cavernoma-Other	-0.545	5.88E-01	ns	1.00E+00	ns
Cavernoma-Unknown	-2.733	8.70E-03	**	4.61E-01	ns
Dual pathology-Other	1.419	1.67E-01	ns	1.00E+00	ns
Dual pathology-Unknown	0.039	9.69E-01	ns	1.00E+00	ns
Other-Unknown	-1.478	1.53E-01	ns	1.00E+00	ns
Temporal-Frontal	3.556	4.15E-04	***	2.20E-02	*
Temporal-Occipital	1.499	1.42E-01	ns	1.00E+00	ns
Temporal-Parietal	3.862	1.78E-04	***	9.44E-03	*
Temporal-Insular	1.902	9.35E-02	ns	1.00E+00	ns
Temporal-Other	2.684	5.49E-02	ns	1.00E+00	ns
Frontal-Occipital	0.307	7.61E-01	ns	1.00E+00	ns
Frontal-Parietal	1.723	8.67E-02	ns	1.00E+00	ns
Frontal-Insular	1.479	1.77E-01	ns	1.00E+00	ns
Frontal-Other	2.421	7.21E-02	ns	1.00E+00	ns
Occipital-Parietal	0.698	4.88E-01	ns	1.00E+00	ns
Occipital-Insular	1.299	2.23E-01	ns	1.00E+00	ns
Occipital-Other	2.304	7.68E-02	ns	1.00E+00	ns
Parietal-Insular	1.072	3.13E-01	ns	1.00E+00	ns
Parietal-Other	2.166	9.42E-02	ns	1.00E+00	ns
Insular-Other	1.279	2.41E-01	ns	1.00E+00	ns
Left-Right	-1.507	1.32E-01	ns	1.00E+00	ns
3T-1.5T	1.466	1.45E-01	ns	1.00E+00	ns
3T-Unknown	0.816	4.15E-01	ns	1.00E+00	ns
1.5T-Unknown	-1.109	2.69E-01	ns	1.00E+00	ns
Isotropy-Anisotropy	-1.559	1.20E-01	ns	1.00E+00	ns
Same year-Following year	-1.017	3.10E-01	ns	1.00E+00	ns
Same year-≥2 years	-1.543	1.24E-01	ns	1.00E+00	ns
Same year-Unknown	-0.843	3.99E-01	ns	1.00E+00	ns
Following year-≥2years	-0.524	6.01E-01	ns	1.00E+00	ns
Following year-Unknown	0.378	7.06E-01	ns	1.00E+00	ns
≥2 years-Unknown	0.991	3.23E-01	ns	1.00E+00	ns

Sig., significance; Sig. corr., significance after bonferroni adjustment; LH, left hemisphere; RH, right hemisphere; MCD, malformation of cortical development; HS, hippocampal sclerosis; LEAT, low grade epilepsy associated tumour; *, p <0.05; **, P< 0.01; ***, p<0.001; ns, not significant

Supplementary Table 10.
MELD-PostOp model subgroup performance across clinical and imaging variables evaluated on the entire cohort (n=1102).

	Number of patients (n)	Percentage (%)	Range	Mean DSC ± SD	Median DSC [IQR]
Age					
Paediatric	496	45.0	0.00 - 0.99	0.88 ± 0.18	0.95 [0.87 - 0.98]
Adult	603	54.7	0.00 - 1.00	0.88 ± 0.18	0.94 [0.87 - 0.98]
Unknown	3	0.3	0.77 - 0.98	0.88 ± 0.10	0.88 [0.83 - 0.93]
Sex					
Male	504	45.7	0.00 - 0.99	0.87 ± 0.19	0.94 [0.86 - 0.98]
Female	465	42.2	0.00 - 1.00	0.89 ± 0.19	0.96 [0.90 - 0.98]
Unknown	133	12.1	0.42 - 0.97	0.88 ± 0.08	0.90 [0.86 - 0.93]
Pathology					
MCD	421	38.2	0.00 - 0.99	0.88 ± 0.18	0.94 [0.86 - 0.98]
HS	190	17.2	0.15 - 0.99	0.94 ± 0.12	0.98 [0.95 - 0.99]
LEAT	194	17.6	0.00 - 0.99	0.88 ± 0.18	0.94 [0.87 - 0.98]
Cavernoma	48	4.4	0.00 - 1.00	0.75 ± 0.33	0.92 [0.66 - 0.98]
Dual pathology	83	7.5	0.00 - 0.99	0.88 ± 0.19	0.96 [0.88 - 0.98]
Other	23	2.1	0.00 - 0.99	0.79 ± 0.29	0.92 [0.82 - 0.95]
Unknown	143	13.0	0.42 - 0.99	0.88 ± 0.08	0.90 [0.86 - 0.93]
Location of resection					
Temporal	633	57.4	0.00 - 1.00	0.91 ± 0.14	0.96 [0.90 - 0.98]
Frontal	304	27.6	0.00 - 0.99	0.87 ± 0.19	0.93 [0.84 - 0.97]
Occipital	39	3.5	0.00 - 0.99	0.85 ± 0.23	0.93 [0.85 - 0.97]
Parietal	112	10.2	0.00 - 0.99	0.82 ± 0.22	0.91 [0.79 - 0.97]
Insular	9	0.8	0.00 - 0.97	0.71 ± 0.31	0.80 [0.65 - 0.93]
Other	5	0.5	0.01 - 0.86	0.46 ± 0.38	0.61 [0.11 - 0.69]
Surgical side					
Left	567	51.5	0.00 - 0.99	0.88 ± 0.19	0.94 [0.87 - 0.98]
Right	535	48.5	0.00 - 1.00	0.89 ± 0.17	0.95 [0.88 - 0.98]
MRI field strength					
3T	845	76.7	0.00 - 1.00	0.89 ± 0.18	0.96 [0.88 - 0.98]
1.5T	124	11.3	0.00 - 0.99	0.86 ± 0.23	0.94 [0.86 - 0.97]
Unknown	133	12.1	0.42 - 0.97	0.88 ± 0.08	0.90 [0.86 - 0.93]
Isotropy					
Isotropic	800	72.6	0.00 - 0.99	0.88 ± 0.18	0.94 [0.86 - 0.98]
Anisotropic	302	27.4	0.00 - 1.00	0.90 ± 0.17	0.96 [0.91 - 0.98]
Time from surgery to postoperative scan					
Same year	332	30.1	0.00 - 0.99	0.87 ± 0.20	0.96 [0.87 - 0.98]
Following year	201	18.2	0.00 - 1.00	0.89 ± 0.18	0.96 [0.87 - 0.98]
≥ 2 years	133	12.1	0.00 - 0.99	0.90 ± 0.15	0.94 [0.87 - 0.98]
Unknown	436	39.6	0.00 - 0.99	0.88 ± 0.16	0.93 [0.87 - 0.98]

MCD, malformation of cortical development; HS, hippocampal sclerosis; LEAT, low grade epilepsy associated tumour; DSC, dice similarity coefficient; IQR, interquartile range

Supplementary Table 11.
Summary of MRI acquisition characteristics of postoperative T1w scans from MELD dataset. Reported parameters include sequence family, contrast status, and slice thickness.

	**Count (n)**
Sequence family	
MPRAGE	178
TFE	140
SPRG/FSPRG	52
SPACE	1
T1w not specified	183
Unknown	415
Contrast status	
Pre/post contrast	40
Non-contrast	18
Unknown	911
Slice thickness	
< 1 mm	98
= 1mm	298
> 1mm	158
Unknown	415

Supplementary Figures

Supplementary Figure 1.
Representative 3*14 visual quality control (QC) image from a case that passed QC.

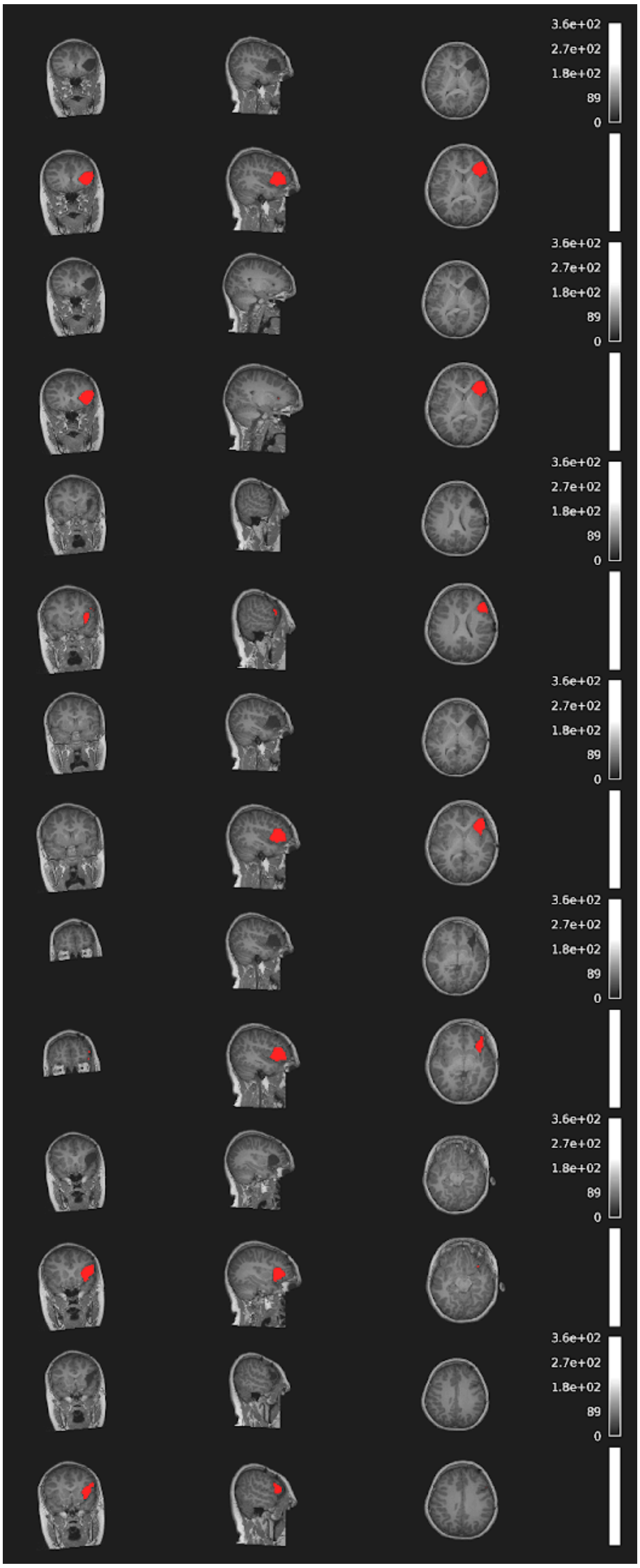

Supplementary Figure 2.
Representative 3*14 visual quality control (QC) image from a case that failed QC. The resection cavity segmentation was incomplete and subsequently corrected through manual editing.

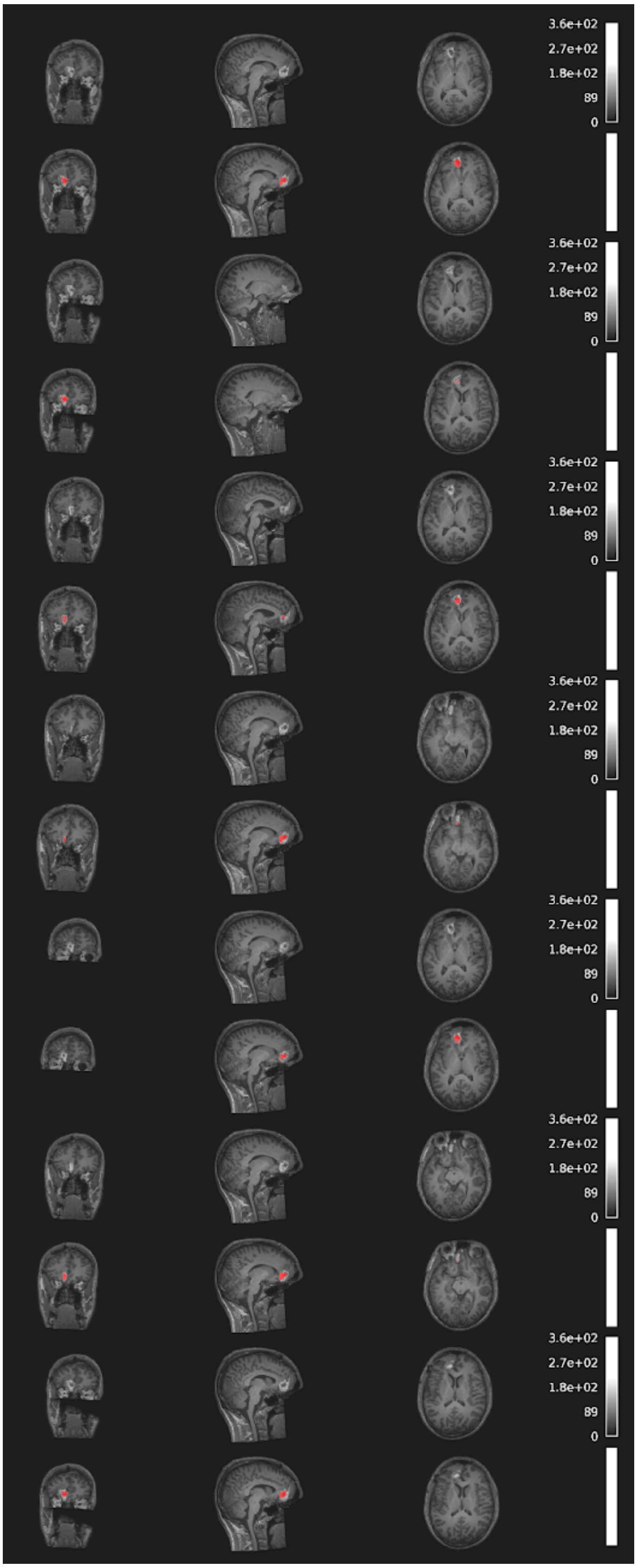

Supplementary Figure 3
Examples of resection segmentations that failed QC. Segmentations were classified as failed if they met one or more of the following criteria: 1) incorrect anatomical location (e.g. off-brain or unrelated region), 2) incomplete coverage of the resection cavity, or 3) inclusion of substantial non-cavity tissue.

Case 1: Incorrect anatomical location
The left panel shows the predicted mask before correction, which segmented non-cavity tissue. The segmentation in the non-cavity tissue was removed during correction. The right panel shows the corrected mask.

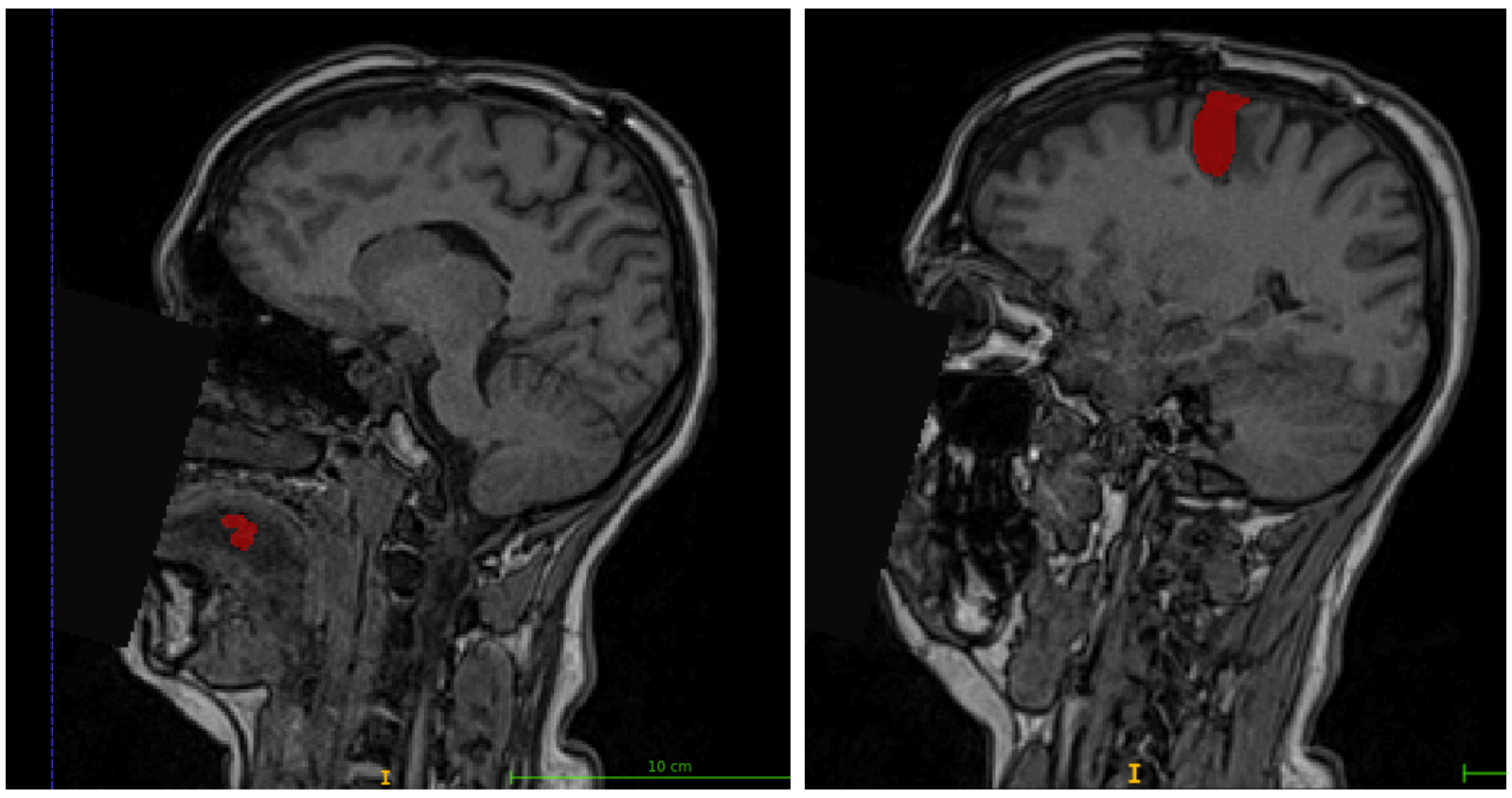

Case 2: Incomplete segmentation
The left panel shows the predicted mask before correction, which did not fully capture the extent of the resection cavity. The right panel shows the corrected mask, which delineates the full cavity.

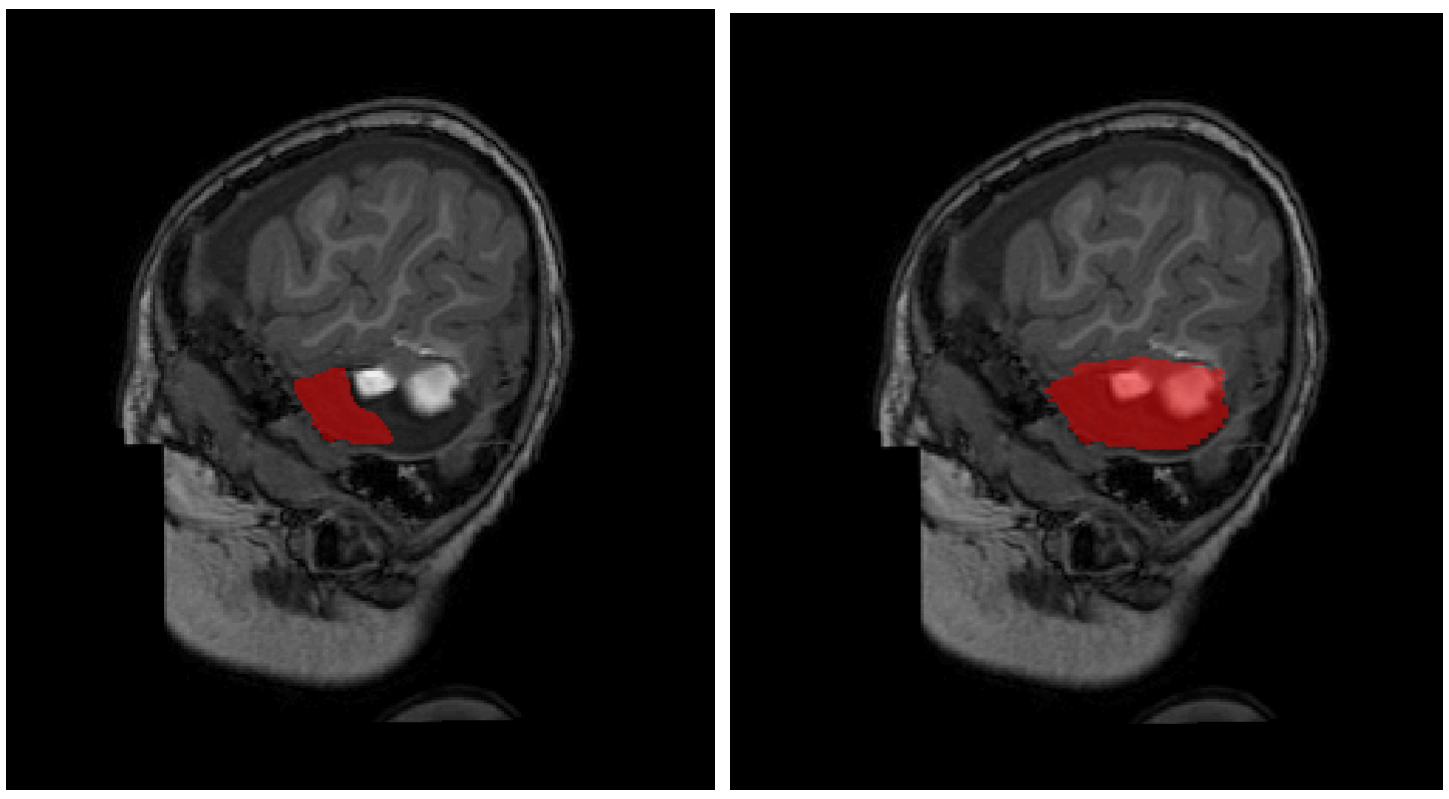

Supplementary Figure 4.
Raincloud plot showing the distribution of DSC for the Prototype Cohort from the MELD (n=152) and EPISURG (n=133) datasets. The median DSC was 0.88 for MELD and 0.90 for EPISURG. A Mann-Whitney U test demonstrated a statistically significant difference (p=0.011), with effect size of 0.157.

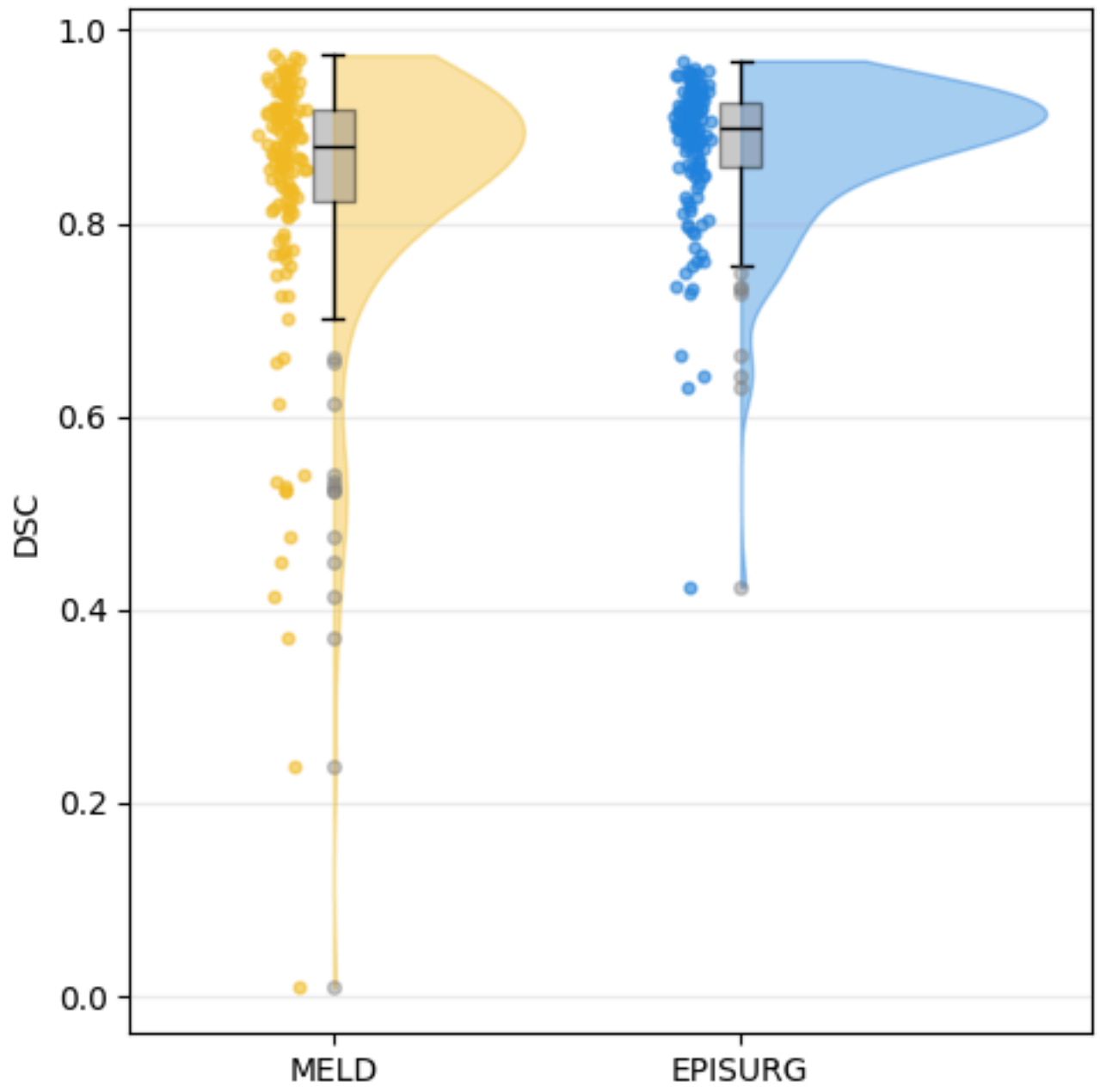

Supplementary Figure 5.
Raincloud plot illustrating change in dice similarity coefficient (DSC) for cases that failed QC after initial inference with the Prototype model (n=86), comparing Prototype and MELD-PostOp performance. Paired values depict within-case changes in segmentation accuracy. Overall DSC increased with MELD-PostOp, indicating improved segmentation performance. Median DSC increased from 0.02 [0.00-0.91] for the Prototype model to 0.65 [0.19-0.90] for MELD-PostOp. This difference was significant (*p* < 0.01).

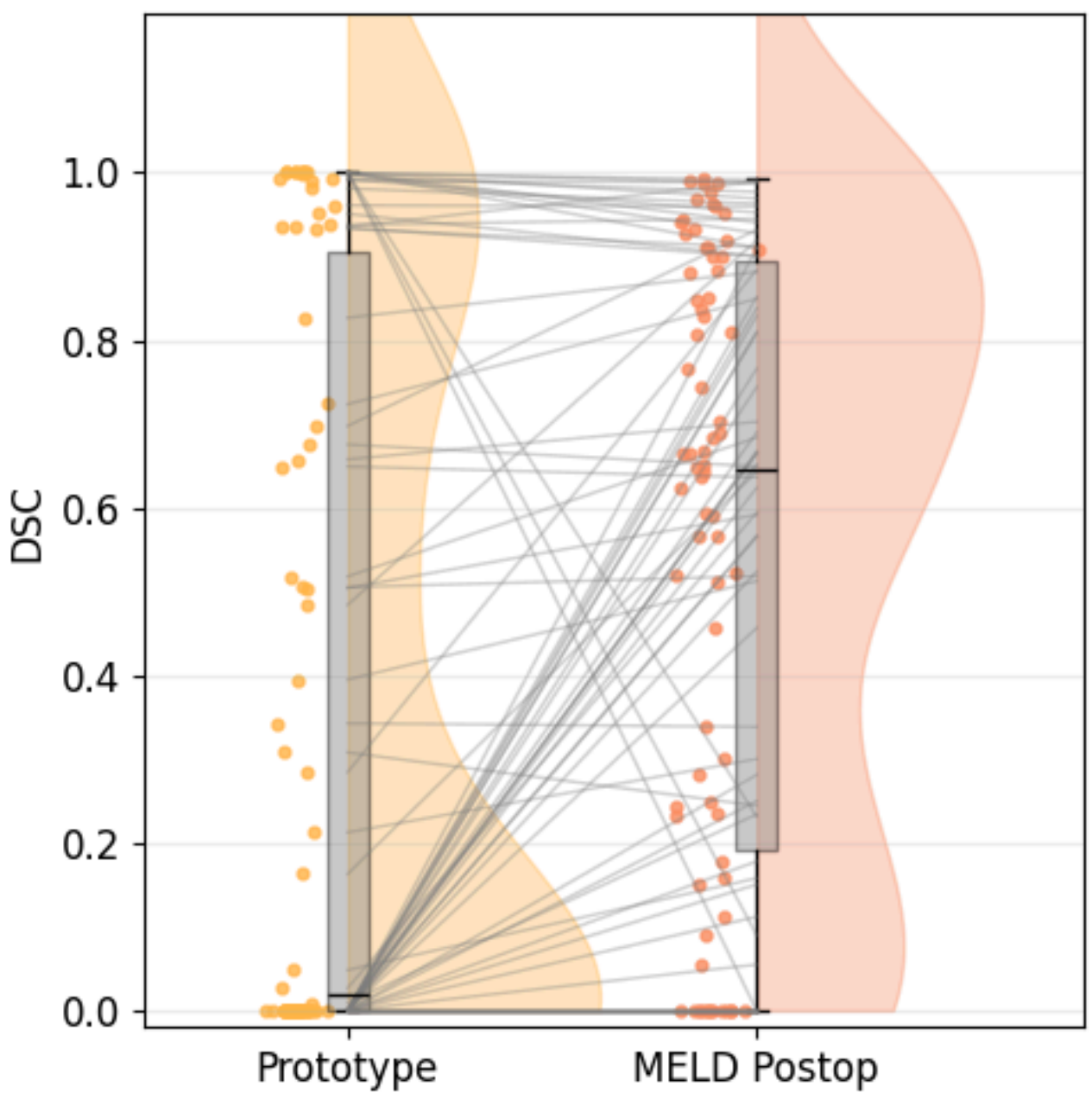

Supplementary Figure 6.
Spearman’s rank correlation between manual resection cavity volume and dice similarity coefficient (DSC) for Combined Test Cohort (n=137), Stratified Test Cohort (n=50), and Independent Test Cohort (n=87).

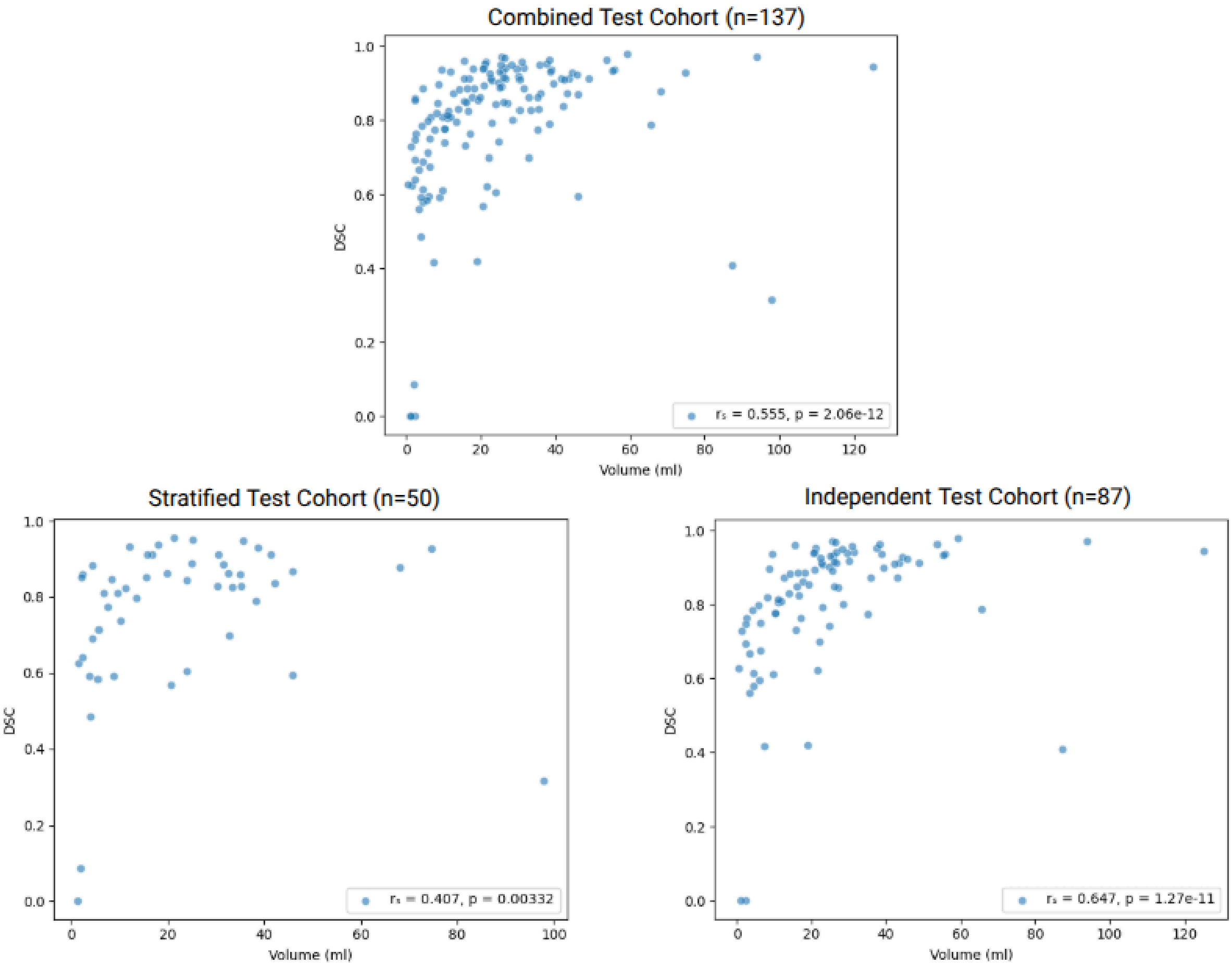

Supplementary Figure 7.
Spearman's rank correlation between manual resection cavity volume and 95th percentile hausdorff distance for Combined Test Cohort (n=137), Stratified Test Cohort (n=50), and Independent Test Cohort (n=87).

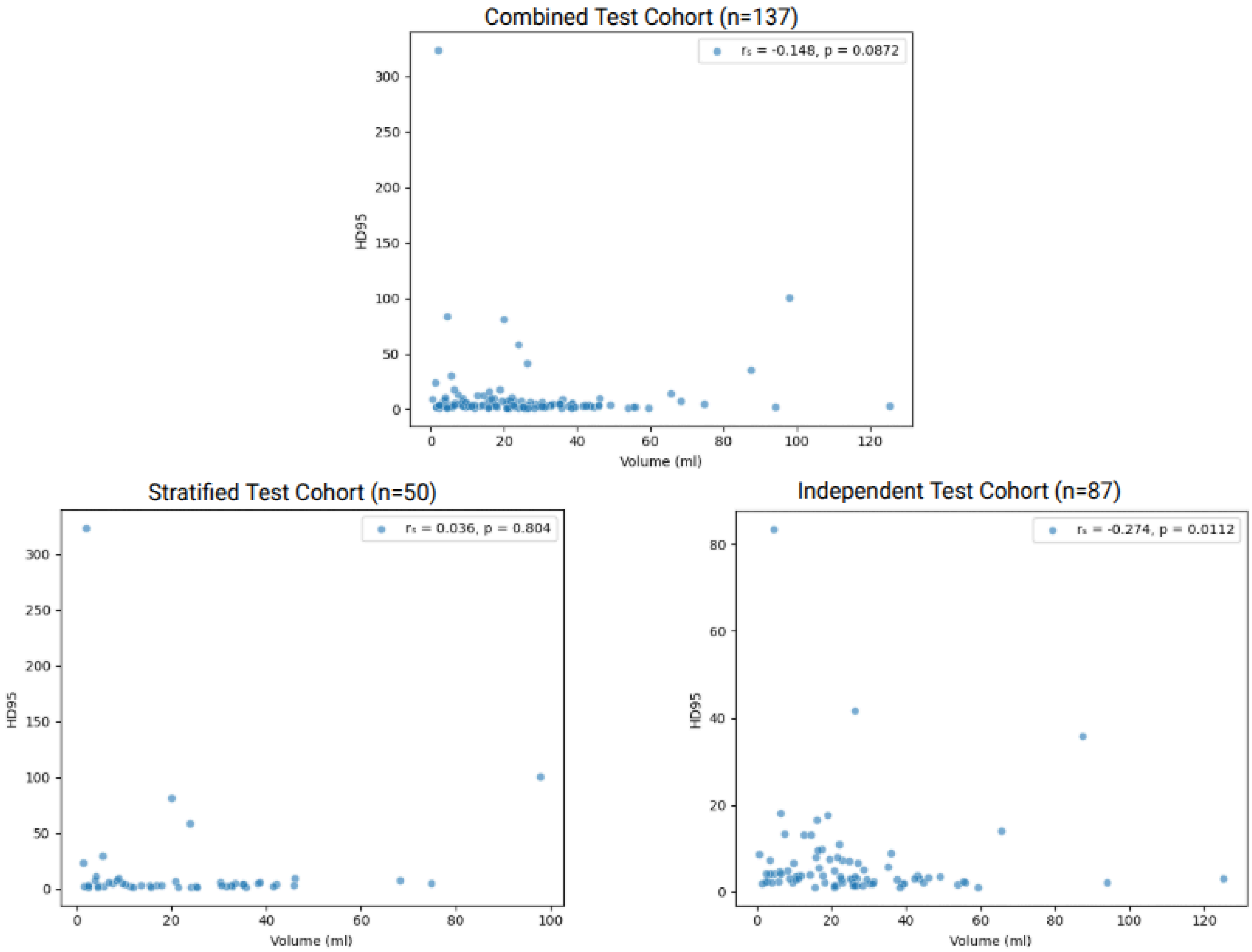

Supplementary Figure 8.
One of two failure cases of MELD-PostOp. This patient was an adult male with cavernoma. Ground truth volume is 1030 mm^3. MELD-PostOp failed to identify the resection cavity, generating no predicted clusters. Epic-CHOP and RESSEG produced false positive segmentations, while ResectVol generated a true positive cluster (DSC=0.354). The second failure case is presented in Figure 3A (Patient 7).

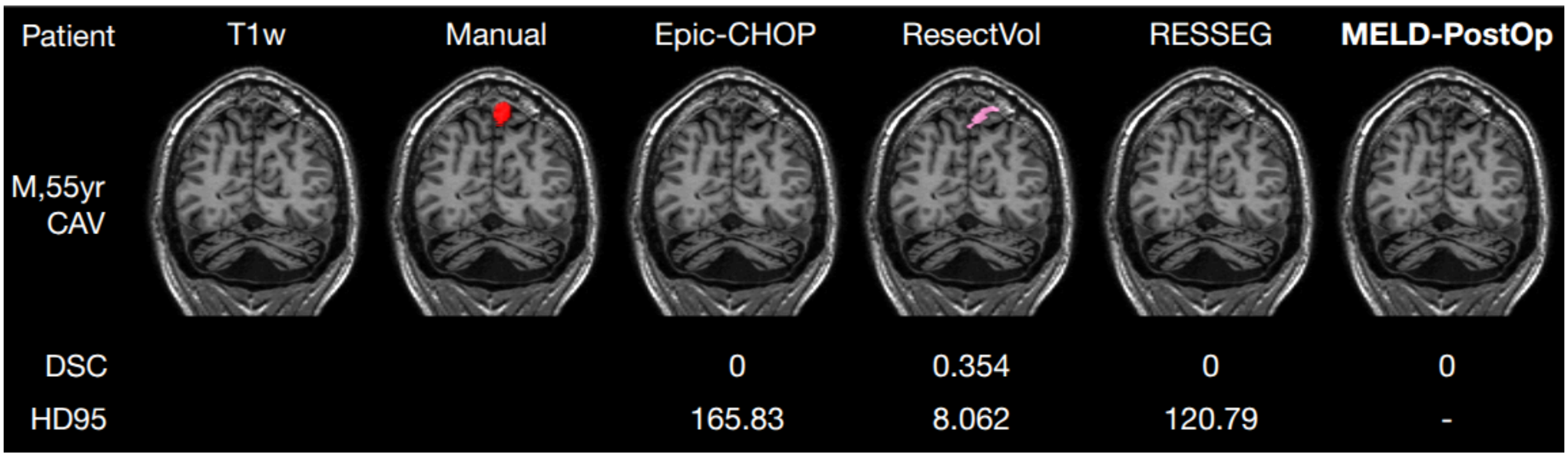

Supplementary Figure 9.
Example of false positive case. The left panel shows the ground truth mask in a 3D view, and in two different slices from a single patient. The right panel shows the MELD-PostOp predictions for the same patient, also in 3D view and in two corresponding slices.
MELD-PostOp predicted two clusters: one true positive and one false positive, resulting in a PPV of 0.5.

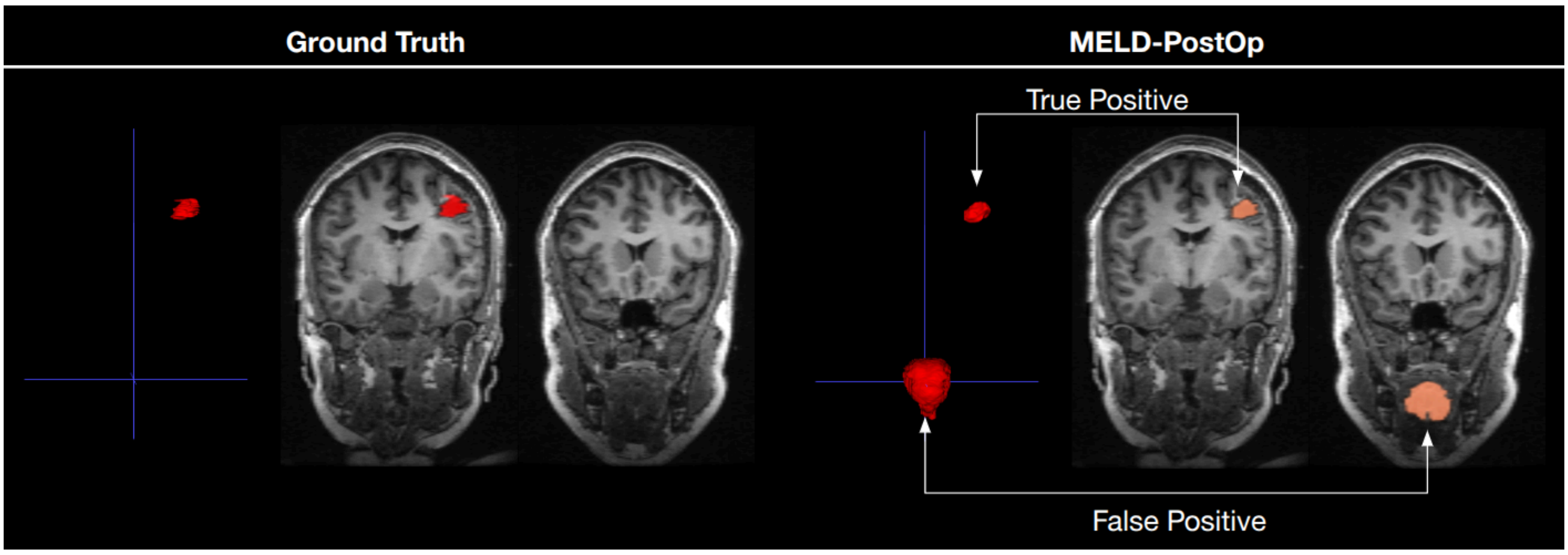

Supplementary Figure 10.
Runtime analysis of Epic-CHOP, ResectVol, RESSEG and MELD-PostOp were performed on the same 10 subjects using identical computational hardware. Epic-CHOP demonstrated a median runtime of 3363.5s, ResectVol 659.0s, RESSEG 4.0s and MELD-PostOp 15.0s. Significant differences were observed between all four models ($p < 0.001$). Mean runtime was 3205s, 612s, 4.2s and 17s, respectively.

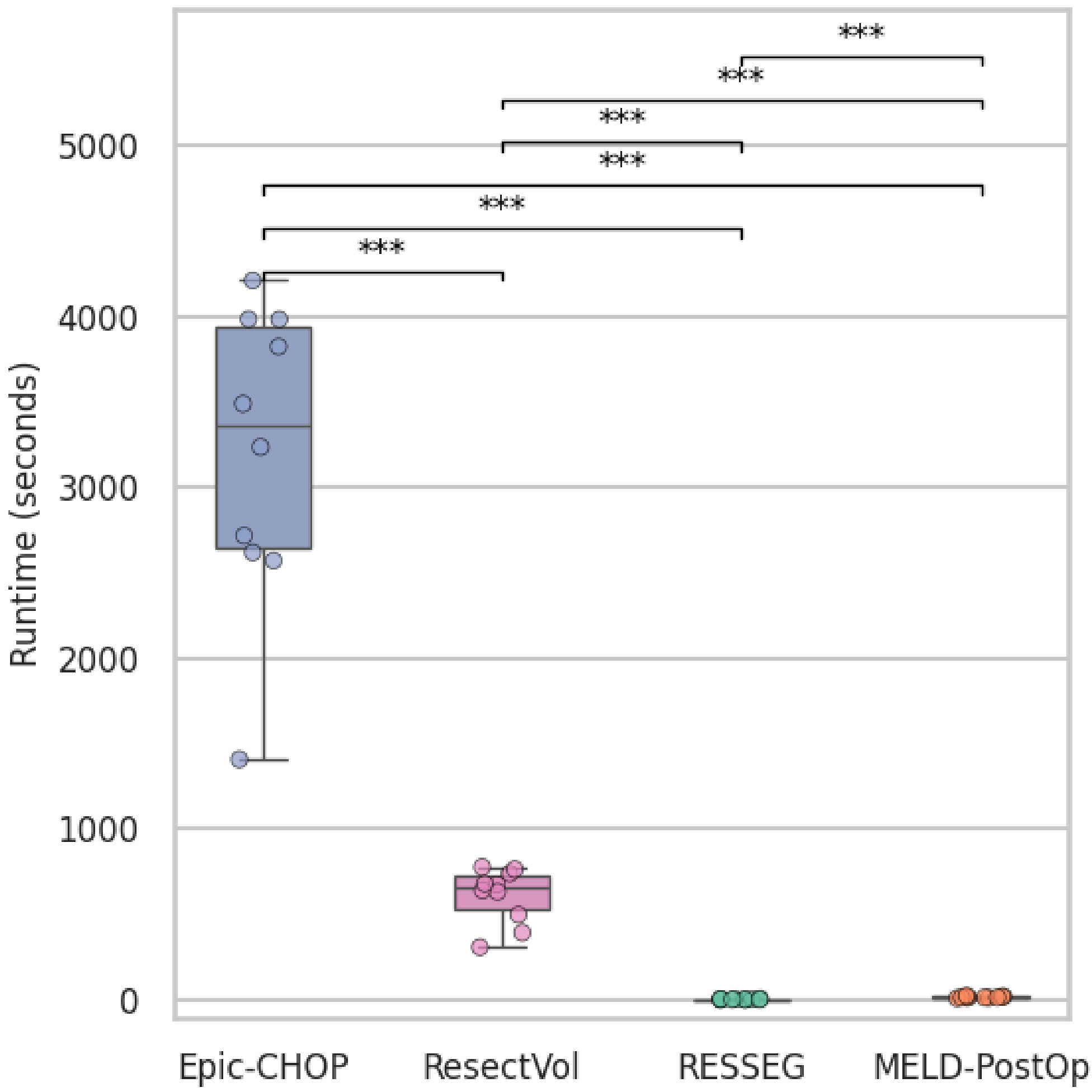

Supplementary Figure 11.
Bland-Altman analysis comparing DSC between MELD-PostOp cross-validation predictions and nnInteractive, and ITK-SNAP segmentations across 40 cases. The central dashed line indicates the mean DSC difference (mean: -0.025), and the dotted lines represent the 95% limits of agreement (-0.160 to 0.109). 37/40 subjects fell within the limits of agreement, with three outliers, indicating overall good agreement between the two segmentation approaches.

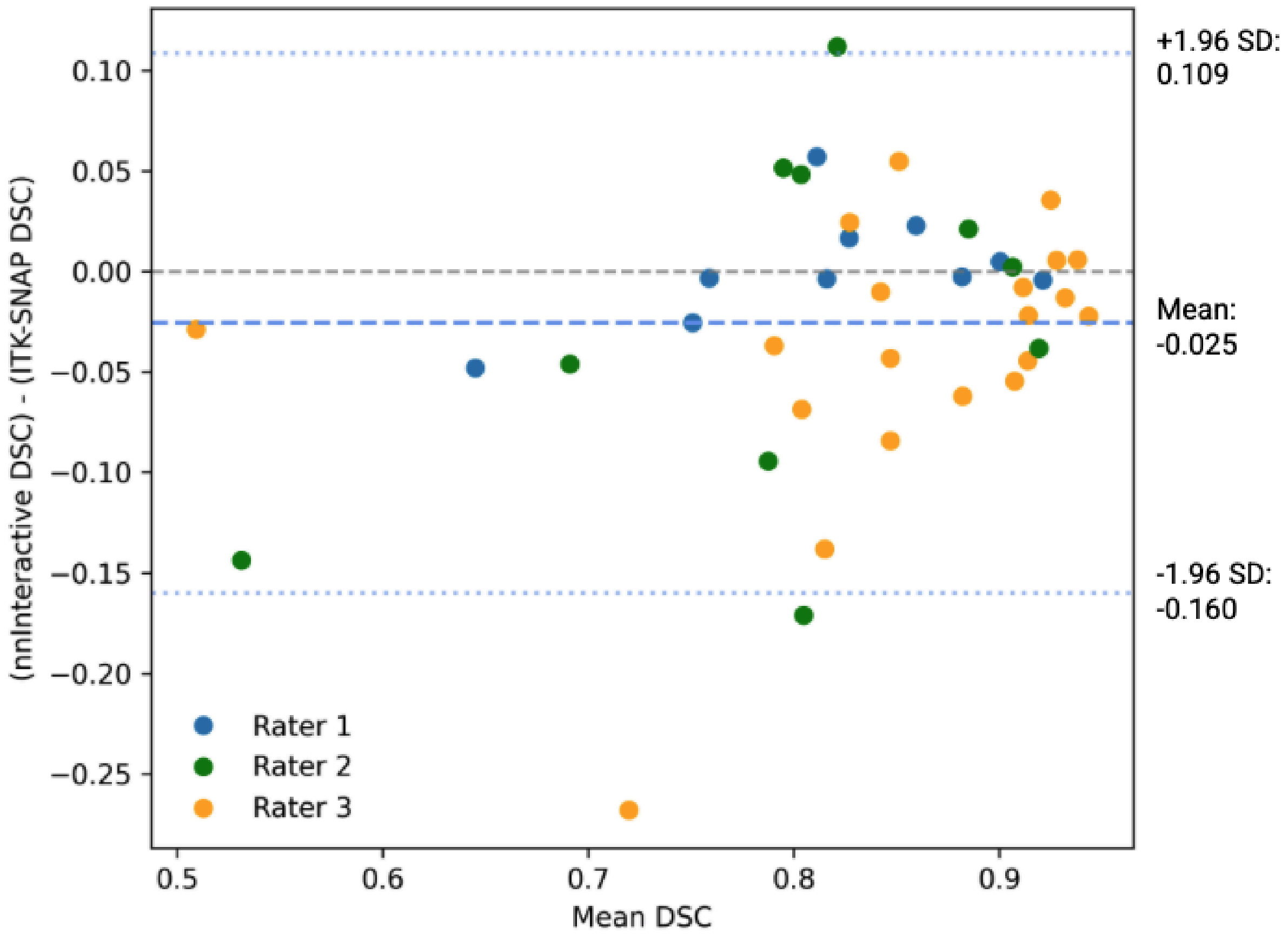

Supplement: Supplementary file 1 [file EPI-67-5901-s001.pdf]
